# Twist-Induced Altermagnetism in a Metallic van der Waals Antiferromagnet

**DOI:** 10.1021/acs.nanolett.6c01391

**Published:** 2026-06-15

**Authors:** Alberto M. Ruiz, Andrei Shumilin, Rafael González-Hernández, José J. Baldoví

**Affiliations:** † Instituto de Ciencia Molecular, Universitat de València, Catedrático José Beltrán 2, 46980 Paterna, Spain; ‡ Departamento de Física y Geociencias, Universidad del Norte, Barranquilla 081007, Colombia

**Keywords:** Altermagnetism, twistronics, Fe_3_GaTe_2_, doping, first-principles calculations

## Abstract

Altermagnetism offers a promising route for next-generation spintronic devices. In two-dimensional (2D) magnets, twist engineering enables its realization by breaking the combined inversion and time-reversal symmetry ( P *T*). Here, by first-principles calculations and symmetry analysis, we demonstrate that twisting the recently synthesized metallic van der Waals antiferromagnet Co-doped bilayer Fe_3_GaTe_2_ (Fe_2_CoGaTe_2_) provides a robust platform for altermagnetism, breaking the P *T* symmetry between opposite spin sublattices. This results in a nonrelativistic *i*-wave altermagnetic state with spin splitting up to 138 meV. Without spin–orbit coupling (SOC) the electronic states remain spin-degenerate along six high-symmetry directions, whereas including SOC preserves degeneracy along the three directions protected by 2-fold rotation axes. Furthermore, we unveil the microscopic mechanisms governing the magnetic behavior in twisted bilayer Fe_2_CoGaTe_2_. Our results establish twist engineering and metallic Fe-based van der Waals antiferromagnets as versatile platforms to realize 2D altermagnetism, with potential for designing high-efficiency ultrathin nanodevices.

## Graphical abstract

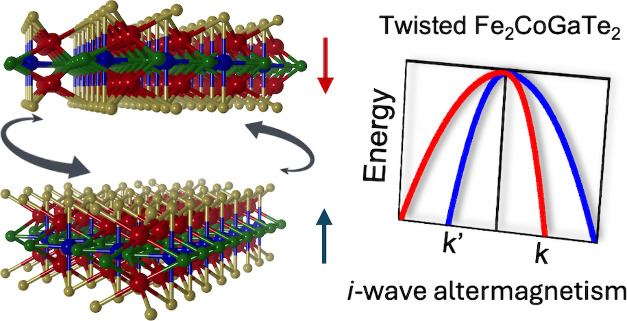

Altermagnetism is a novel magnetic state characterized by the coexistence of spin-polarized electronic bands and zero net magnetization, distinguishing it from conventional ferromagnets and antiferromagnets.^1–4^ This combination offers significant advantages for next-generation spintronic applications, such as the spin Hall effect and magneto-optical Kerr effect, primarily due to the absence of stray fields and the promise of ultrafast spin dynamics. Unlike conventional antiferromagnets, in which opposite spin sublattices are related by translational (τ) or inversion ( P ) symmetry, the altermagnetic (AM) state requires that these sublattices be connected via specific rotational ( C ) or mirror ( M ) symmetry operations. Consequently, the realization of altermagnetism requires the breaking of the combined P and time-reversal (*T*) symmetries, known as P *T*, which enables spin splitting while preserving specific C or M symmetries that enforce the characteristic alternation of spin polarization in momentum space.^5–8^

Two-dimensional (2D) magnetic materials provide an attractive platform for realizing altermagnetism, as their tunable lattice symmetry and magnetic interactions allow for fine control of their properties. However, many well-studied van der Waals (vdW) A-type antiferromagnets, such as CrI_3_ and CrSBr, possess τ or P symmetries between opposite spin sublattices.^9,10^ This enforces conventional antiferromagnetic (AF) behavior and forbids the momentum-space spin splitting required for an AM state.^11–13^ Among the existing 2D vdW magnetic materials, the recently synthesized vdW itinerant ferromagnet Fe_3_GaTe_2_ has attracted significant attention due to its above-room-temperature magnetism (T_C_ = 350–380 K), metallic character and out-of-plane anisotropy.^14–21^ Indeed, in the context of altermagnetism, metallic altermagnets are particularly appealing due to the possibility of direct electrical control and efficient manipulation of spin currents via applied electric fields.^22^ Nevertheless, altermagnetism is prohibited in Fe_3_GaTe_2_ given that (i) it exhibits a FM ground state and (ii) spin sublattices of adjacent layers are connected through P symmetry. Partial substitution of Fe with Co in (Fe_1–x_Co_*x*_)_3_GaTe_2_ has recently been experimentally demonstrated to induce a transition from ferromagnetic (FM) to AF ground state, solving (i), whereas (ii) is still present, thus preventing AM spin splitting.^23–25^

As a consequence of the lack of vdW 2D magnetic materials that naturally host AM order, several theoretical strategies have been proposed to induce altermagnetism. These include the application of a gate-induced out-of-plane electric field, ligand substitution or creation of heterostructures.^26–28^ Another well-stablished route is the realization of Janus 2D magnetic materials, in which the two faces of the material are chemically different, inducing an out-of-plane asymmetry within the layer.^29–32^ These approaches break the combined P *T* symmetry while preserving selected symmetry operations relating opposite spin sublattices.

Besides these approaches, twistronics—the tuning of the relative twist angle between layers in vdW heterostructures—has emerged as an unprecedented platform for exploring novel electronic and magnetic states since 2018.^33–35^ In the context of altermagnetism, it has been proposed that AM order can be induced upon rotation between two layers.^36–38^ However, most materials explored to date exhibit semiconducting behavior and some of these works rely on the assumption that antiferromagnetism present in the parent nonrotated compounds is preserved upon twisting. Consequently, identifying metallic vdW materials that can retain AF interlayer coupling under twisting remains a central challenge for the realization of twist-induced altermagnetism in vdW materials.

Here, we overcome these limitations by demonstrating that metallic Co-doped bilayer Fe_3_GaTe_2_ hosts robust AM order upon twisting. Using first-principles calculations, we show that Co doping drives Fe_3_GaTe_2_ from a FM to an AF state, consistent with recent experimental reports. Importantly, our symmetry analysis reveals that upon twisting, the AF interlayer coupling is preserved, while the rotation breaks the P *T* symmetry that connects opposite spin sublattices. This breaking gives rise to a nonrelativistic spin-split electronic band structure with a maximum splitting of 138 meV around the Fermi level. Our results position twistronics as a versatile platform for inducing altermagnetism in twisted 2D magnetic materials, as well as the use of Fe_3_GaTe_2_ for efficient spintronic applications.

Fe_3_GaTe_2_ is a vdW magnetic material that crystallizes in a hexagonal layered structure belonging to the centrosymmetric space group *P*6_3_/*mmc* (No. 194).^14^ Each layer comprises five atomically distinct sublayers, where the outermost planes are formed by Te, followed by two Fe sublayers, while the central plane is constituted by Ga and Fe atoms (Figure 1a). Between adjacent Fe_3_GaTe_2_ layers, there is an inversion center and thus the system shows P symmetry. We first perform first-principles calculations on bilayer Fe_3_GaTe_2_, for which the symmetry is reduced to *P*3̅*m*1 (No. 164).^39^ The optimized lattice parameters using the GGA scheme are *a* = *b* = 4.03 Å and the computed averaged magnetic moment for Fe atoms is 2.03 μ_B_ (Figure S1 and Table S1). The material exhibits FM coupling between layers, which is determined by computing the interlayer exchange coupling (J_int_). It is defined as J_int_ = E_AF_ – E_FM_ and is found to be 1.18 meV/Fe, correctly capturing the experimentally observed magnetic ground state.^14^ This spin alignment gives rise to a Zeeman-type splitting of the electronic bands, as illustrated in Figure 1a. Furthermore, Fe_3_GaTe_2_ exhibits out-of-plane anisotropy with spins aligned along the *c* axis, quantified by computing the magnetic anisotropy energy (MAE), defined as the energy difference between in-plane and out-of-plane spin orientations (MAE = E_∥_ – E_⊥_), resulting in MAE = 0.45 meV/Fe.^40^ Figure S2 depicts the computed electronic band structure of bilayer Fe_3_GaTe_2_, showing multiple bands crossing the Fermi level, where electron and hole pockets are observed around the K and Γ points, respectively, in agreement with ARPES measurements and previous theoretical reports.^19,20,41^

**Figure 1:**
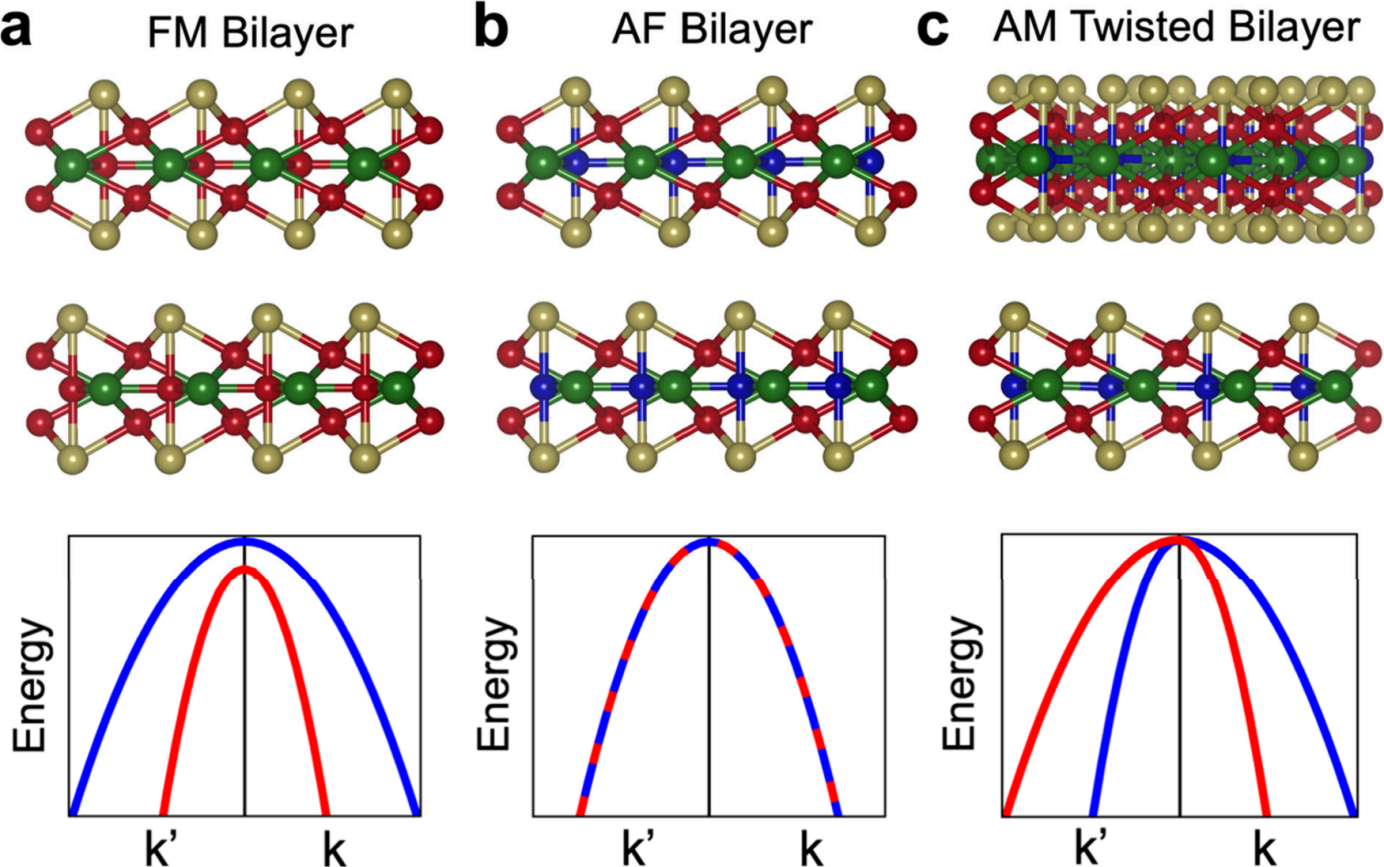
(a) Lateral view of bilayer Fe_3_GaTe_2_ with its corresponding schematic FM-like band structure. (b) Lateral view of bilayer Fe_2_CoGaTe_2_ and its correspondent schematic degenerate AF band structure. (c) Lateral view of 21.79° twisted bilayer Fe_2_CoGaTe_2_, where the noncentrosymmetric structure gives rise to AM spin splitting. Color code: Fe (dark red), Co (dark blue), Ga (green), and Te (yellow). Blue (red) color in the band structure indicates the projected spin up (down) states.

We next consider the substitution of Fe with Co in (Fe_1–*x*_Co_*x*_)_3_GaTe_2_, focusing on a doping level of *x* = 1/3, corresponding to the composition Fe_2_CoGaTe_2_. This concentration is selected based on recent experimental reports showing that Co doping induces interlayer antiferromagnetism in (Fe_1–*x*_Co_*x*_)_3_GaTe_2_ for 0.15 < *x* < 0.40.^23–25^ Among the possible substitutional arrangements, the energetically most favorable configuration is the one in which Co atoms occupy the Fe sites in the central layer (Figure 1b; Figure S3 and Table S2),^42^ retaining the centrosymmetric *P*3̅*m*1 space group.^25^ For Fe_2_CoGaTe_2_, the computed magnetic moments are 2.56 μ_B_/Fe and 0.88 μ_B_/Co and the interlayer exchange coupling becomes strongly AF, where J_int_ = −2.44 meV per magnetic atom. The out of plane anisotropy is preserved, showing an enhanced value of MAE = 1.44 meV per magnetic atom. Spin–space–group (SSG) analysis reveals that Fe_2_CoGaTe_2_ belongs to a type-III AF group that contains the antiunitary generator [−1||−1], where in the notation [S||R] the first entry S denotes the spin-space operation (−1 corresponds to spin inversion) and the second entry R specifies the associated real-space operation (−1 is spatial inversion). This [−1||−1] operation connects the two opposite Fe (or Co) spin sublattices and, because it squares to −1, it enforces a Kramers-like double spin degeneracy of energy bands in the full Brillouin zone, resulting in conventional metallic AF band structure (Figure 1b and Figure S4). As in Fe_3_GaTe_2_, we note the presence of electron (around K) and hole (around Γ) pockets. Compared to Fe_3_GaTe_2_, these pockets are shifted downward due to the additional electrons introduced by Co atoms (Figure S4).

Then, we analyze the Fe_2_CoGaTe_2_ twisted bilayer upon a rotation angle of 21.79°, showing in-plane lattice parameters *a* = *b* = 10.71 Å (Table S3). This twist angle is selected since it breaks the P *T* symmetry while inducing a zero strain in both sublattices,^43^ and it gives rise to an AM spin splitting in the nonrelativistic limit (Figure 1c). The magnetic moments of Fe and Co atoms remain essentially unchanged relative to the untwisted structure, whereas the interlayer coupling retains its AF character, with J_int_ = −0.54 meV per magnetic atom. The reduced magnitude of J_int_ with respect untwisted Fe_2_CoGaTe_2_ arises from the increased spacing between magnetic centers, which diminishes the orbital overlap mediating the AF exchange interactions. Specifically, the interlayer separation increases from 2.94 Å in untwisted bilayer Fe_2_CoGaTe_2_ to 3.25 Å upon rotation. Nevertheless, the interlayer coupling remains stronger than in other 2D vdW magnets such as CrSBr (J_int_ ≈ −0.1 to −0.2 meV/Cr), and comparable to bilayer CrPS_4_ (J_int_ ≈ −0.50 meV/Cr).^44–46^ Importantly, calculations performed using the LDA functional confirm the robustness of the interlayer AF coupling, yielding J_int_ = −0.86 meV per magnetic atom. Additionally, twisted Fe_2_CoGaTe_2_ shows spins along the out-of-plane *c* axis, where MAE = 1.64 meV/magnetic atom. While noncollinear magnetic textures can arise in twisted vdW magnets at small twist angles, twisted Fe_2_CoGaTe_2_ lies in the large-angle regime (θ = 21.79°) within a small supercell (*a* = *b* = 10.71 Å), where such effects are expected to be suppressed. Indeed, recent experiments in twisted Fe_3_GeTe_2_ and CrI_3_ show that skyrmion phases and noncollinear textures are restricted to small angles and disappear at larger rotations (≳1–2°).^47,48^

In twisted-Fe_2_CoGaTe_2_, the relative rotation between layers breaks the global [−1||−1] ( P *T* symmetry). As a consequence, the two opposite spin sublattices are no longer related by P *T* symmetry, but instead are connected through 180° spatial rotations combined with spin inversions, encoded in the SSG operations: [−1||2_120_], [−1||2_1–10_], and [−1||2_210_]. In addition, collinear magnets exhibit the symmetry *T*[−1||1], which combines time-reversal with a spin-flip. Meanwhile, the unitary operator [1||3_001_] connects site within the same spin sublattice. These symmetry operations act in the real space and are illustrated in Figure 2a (see also Figure S5 for details). Their combined effect constrains the electronic structure in reciprocal space, leading to a characteristic spin splitting pattern in which the spin projection alternates every 30° in momentum space (Figure 2b). As a result, spin splitting becomes symmetry-allowed in the nonrelativistic limit, and the corresponding reduction in SSG operations identifies the twisted structure as an *i*-wave altermagnet. This behavior is not limited to the 21.79° rotation. Other additional twist angles also give rise to *i*-wave altermagnetism (e.g., 13.17°, 27.79°, 32.21°, 38.21° or 46.83°),^36,37,49^ since they preserve *C*_*2*_ symmetry between opposite spin sublattices while maintaining C_3*z*_ symmetry within the same spin sublattice (Table S4 and Figure S6), as first introduced by Liu et al.^36^ Notably, twist angles in this range have already been experimentally realized in related hexagonal vdW materials such as graphene, MoS_2_, and CrI_3_.^34,50–52^

**Figure 2:**
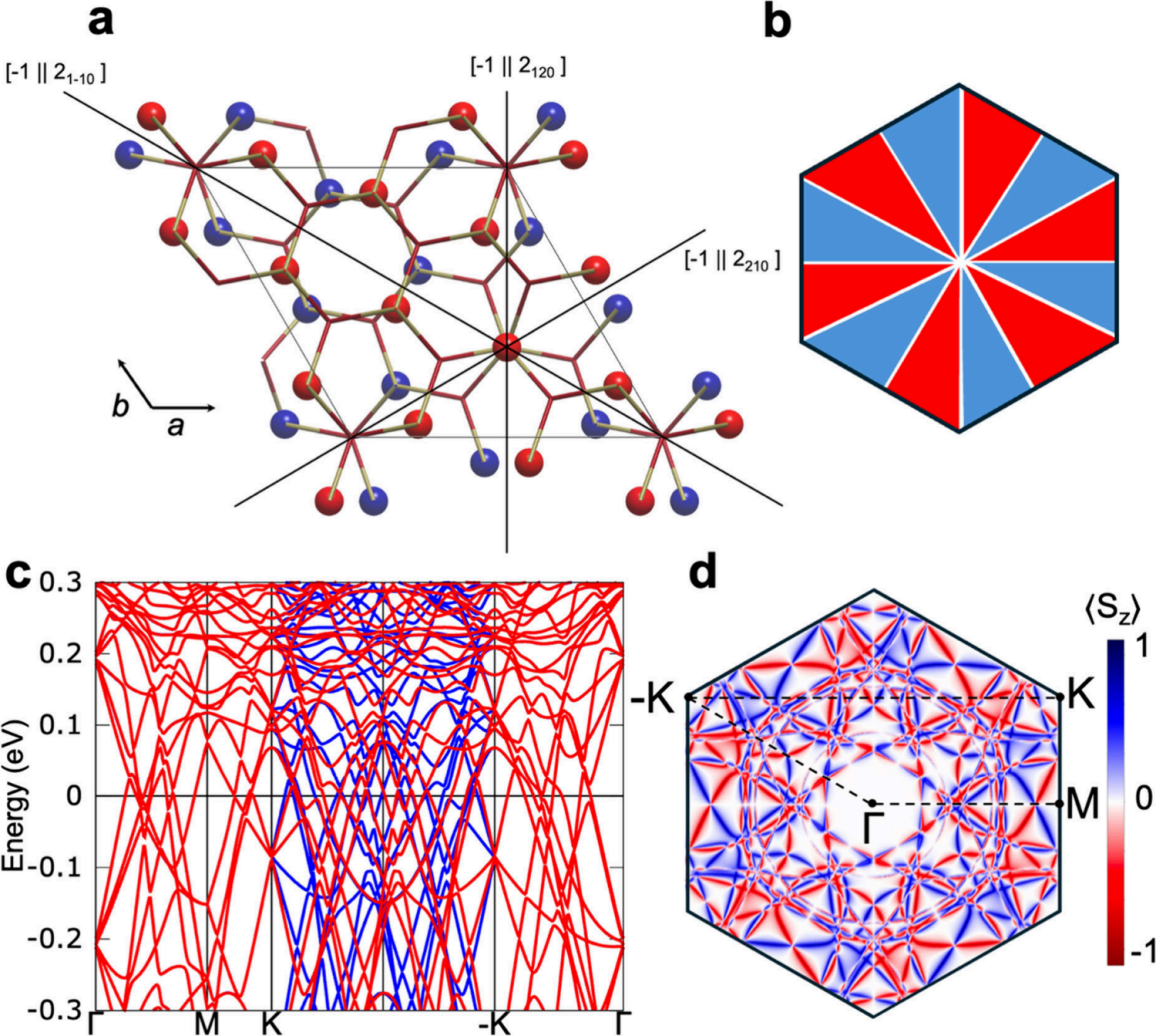
(a) Top view of twisted bilayer Fe_2_CoGaTe_2_, where the blue and red balls represent Co atoms from the bottom and top layer, respectively. For clarity, we only represent Co atoms of the material. Additionally, we show the symmetry operations relating opposite Co spin sublattices. (b) Schematic visualization of the spin-split bands according to the spin symmetry operations on the first Brillouin zone. (c) Nonrelativistic electronic band structure of twisted bilayer Fe_2_CoGaTe_2_ along the high-symmetry path Γ–M–K–(−)­K−Γ. Blue (red) color in the band structure indicates spin up (down) states. (d) Spin-resolved Fermi surface at the Fermi level. The color scale encodes the normalized spin expectation value ⟨S_*z*_ (k)⟩, with blue and red indicating opposite spin polarizations in the nonrelativistic limit.

The AM character of twisted Fe_2_CoGaTe_2_ is clearly reflected in the electronic band structure (Figure 2c). The material exhibits metallic character, with several bands crossing the Fermi level. Spin-degenerate bands are observed along the Γ–M–K path, whereas spin splitting emerges along the K–(−)K direction, showing a more pronounced effect in the valence bands than in the conduction bands. Within the studied energy range, the maximum spin splitting reaches 138 meV for bands located approximately 0.2 eV below the Fermi level, while at the Fermi energy the largest splitting is 70 meV. The calculated electronic band structures for the additional twist angles are provided in Figure S7, confirming the presence *i*-wave altermagnetism.

The spin-resolved Fermi surface shown in Figure 2d exhibits a set of star-like sheets arranged with 6-fold symmetry. The Fermi surface regions are spin-polarized, but their contributions to the total magnetization vanish due to the symmetry-enforced compensation. The Fermi surface topology exhibits the characteristic AM pattern predicted by the symmetry analysis: the spin polarization alternates every 30°, reflecting the momentum-dependent spin splitting imposed by the underlying SSG operations. In addition, the overall Fermi surface pattern retains a 120° rotational symmetry, consistent with the [1||3_001_] symmetry of twisted bilayer Fe_2_CoGaTe_2_.

In the presence of SOC, the material belongs to the noncentrosymmetric magnetic space group *P*3_1_2 (#149.21). In this case, the 2-fold rotations act simultaneously on spatial coordinates and spin directions, without requiring an additional time-reversal operation. This symmetry forbids spin degeneracy perpendicular to the rotation axis, and in particular along the out-of-plane direction (see Figure S8 for details). As a result, the electronic band structure along the directions of the 2-fold rotation axes remains degenerate, even when SOC is included. Figure 3 shows the electronic states at the Fermi level calculated for the twisted Fe_2_CoGaTe_2_ bilayer without and with SOC, respectively. Consistent with our symmetry analysis, the states are spin-degenerate along all six directions in the absence of SOC. When SOC is included, the spin degeneracy is preserved along the directions of the 2-fold rotation axes (indicated by green lines), but lifted along the perpendicular directions (dashed black lines), where no symmetry protection is provided by the magnetic space group #149.21. This behavior is further reflected in the computed electronic band structure, showing that SOC induces a strongly anisotropic response (Figure S9). Specifically, the bands remain degenerate along Γ–M, whereas SOC-driven spin splitting appears along the (−)­K−Γ direction, reaching values of ∼ 45 meV around the Fermi level. In both directions, SOC further induces an energy shift due to the strong spin–orbit character of Te atoms.

**Figure 3:**
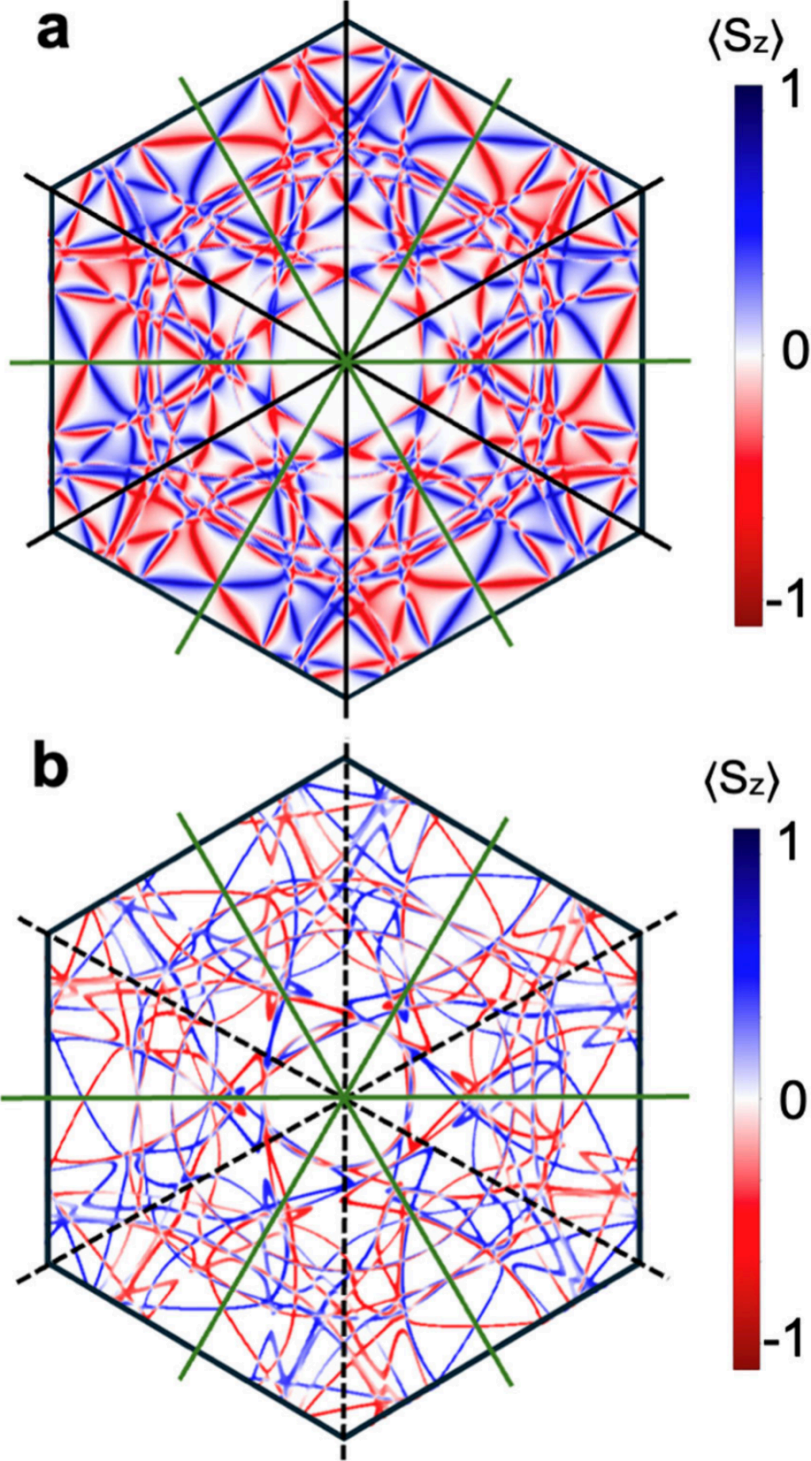
Spin-resolved Fermi surface together with the planes along the directions of the 2-fold rotation axes (green lines) and perpendicular to them (black lines) for twisted Fe_2_CoGaTe_2_ (a) without and (b) with SOC effects. Blue and red lines represent the normalized expectation value of the spin component ⟨S_*z*_ (k)⟩.

These results show that magnetic anisotropy and spin orientation are essential for correctly describing altermagnetism in twisted bilayers. Due to its out-of-plane anisotropy, Fe_2_CoGaTe_2_ retains zero net magnetization upon twisting, inducing an AM state. Note that 90°-twisted CrSBr, although theoretically proposed as an altermagnet in the nonrelativistic limit,^36,49^ experimental findings have reported that spins remain aligned along the easy *b*-axis of each layer.^53–55^ This results in an orthogonal spin arrangement with a global FM state, precluding altermagnetism.

Additionally, given that Co-doped Fe_3_GaTe_2_ samples may contain Fe/Co chemical disorder under experimental conditions, we further assess the robustness of altermagnetism against random Fe/Co substitutions. Our calculations show that some AM features remain robust at low disorder levels (∼10%), while those are progressively weakened at higher concentrations (Figures S10–S12). Nevertheless, the system retains nonrelativistic spin splitting together with nearly zero net magnetization, evolving toward a fully compensated ferrimagnetic state (Table S5). Notably, all disordered configurations are energetically less favorable than the ordered altermagnetic structure considered in the text (Table S5), in agreement with the centrosymmetric space group *P*6_3_/*mmc* observed in recent experimental reports for Co-doped bulk Fe_3_GaTe_2_.^25^

Finally, we examine whether twisting pristine bilayer Fe_3_GaTe_2_ can host an AF ground state and thereby give rise to altermagnetism. We find that, upon twisting, Fe_3_GaTe_2_ remains FM with an interlayer exchange coupling of J_int_ = 0.61 meV/Fe. The magnitude of J_int_ is reduced compared to the untwisted case (J_int_ = 1.14 meV/Fe) due to the increased interlayer spacing (3.4 Å vs 2.94 Å). Consequently, the electronic bands exhibit FM spin splitting throughout the Brillouin zone (Figure S13).

The exchange interactions in twisted Fe_2_CoGaTe_2_ are obtained using a spin Hamiltonian of the form

$$H=-\underset{i\neq j}{\sum }J_{\mathrm{ij}}{\mathrm{S⃗}}_{i}\cdot {\mathrm{S⃗}}_{j}$$

where J_ij_ represent the isotropic exchange interactions, S_i_ and S_j_ are the magnetic moments of different sites and normalized to 1.

The J_ij_ for each layer can be divided into two main categories. The first comprises interplane couplings, which include the interactions between Fe_1_ and Fe_2_, denoted as *J*_Fe1–Fe2_, as well as *J*_Fe1–Co_ (between Fe_1_ and Co) and *J*_Fe2–Co_ (Fe_2_ and Co). In bulk and monolayer Fe_2_CoGaTe_2_, *J*_Fe1–Co_ and *J*_Fe2–Co_ are equivalent given that Fe_1_ and Fe_2_ share identical local environment. This equivalence is lost in the bilayer, where Fe_1_ faces vacuum on one side, while Fe_2_ is adjacent to the vdW gap and the neighboring Fe_2_CoGaTe_2_ layer (Figure 4a). The second category corresponds to in-plane exchange, involving atoms located at the same z coordinate, including *J*_Fe1–Fe1,_ J_Fe2–Fe2_ and *J*_Co–Co_. As shown in Figure 4b, the selected exchange interactions decrease with increasing distance and vanish at 16 Å. For clarity, Figure 4b displays *J*_Fe1–Fe2_, *J*_Fe1–Co_, *J*_Fe1–Fe1_ and *J*_Co–Co_, since the corresponding interactions *J*_Fe2–Co_ and *J*_Fe2–Fe2_ are almost identical to *J*_Fe1–Co_ and *J*_Fe1–Fe1_ (Figure S14), respectively, owing to the nearly equivalent magnetic moments of Fe_1_ and Fe_2_ (2.59 μ_B_ vs 2.54 μ_B_). We observe that the interplane couplings are strongly FM, with *J*_Fe1–Fe2_ and *J*_Fe1–Co_ exhibiting values of 56.8 and 10.7 meV, respectively. In contrast, the in-plane interactions are AF, where *J*_Fe1–Fe1_ = −1.3 meV and *J*_Co–Co_ = −0.8 meV, resulting in a frustrated spin–lattice. This behavior resembles that of Fe_3_GeTe_2_, where the strong ferromagnetism arising from interplane exchange is diminished due to the AF character of in-plane interactions.^19,56^ A direct comparison of the exchange couplings for twisted and untwisted Fe_2_CoGaTe_2_ (Figure S15) shows that those are nearly identical, indicating that twisting primarily affects interactions between adjacent layers.

**Figure 4:**
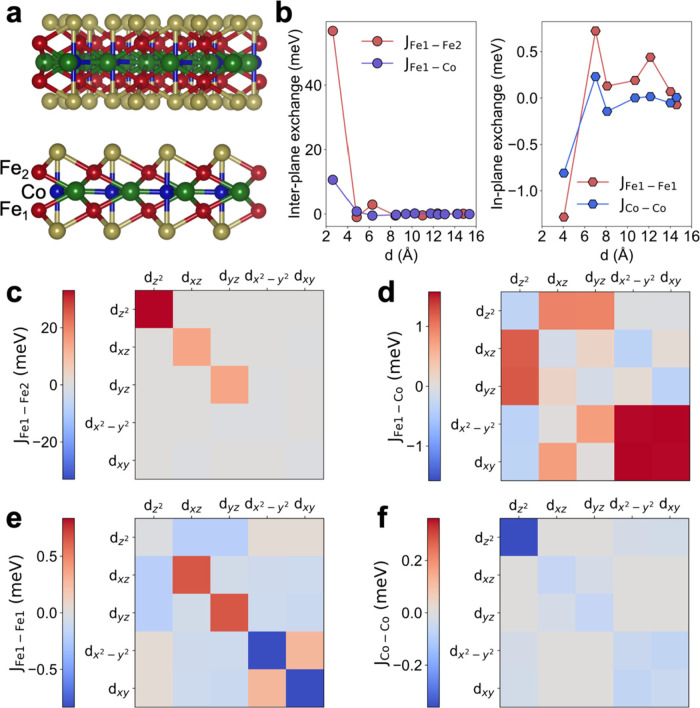
(a) Lateral view of twisted bilayer Fe_2_CoGaTe_2_ labeling the Fe_1_, Fe_2_ and Co atom within one layer. (b) Interplane exchange interactions J_Fe1–Fe2_, J_Fe1–Co_ (left) and in-plane couplings J_Fe1–Fe1_ and J_Co–Co_ (right) for twisted bilayer Fe_2_CoGaTe_2_. (c–f) Orbital-resolved contribution to J_Fe1–Fe2_, J_Fe1–Co_, J_Fe1–Fe1_ and J_Co–Co_, respectively.

The contrasting FM and AF character of the interplane and in-plane couplings arises from the orbitals stabilizing the long-range magnetic order in this material. This is analyzed microscopically through the orbital-resolved contributions to the exchange (Figure 4c–f). We find that the FM character of J_Fe1–Fe2_ is mainly due to the contribution of d_*z* ^2^–_d_*z* ^2^_, d_*xz*–_d_*xz*_ and d_*yz*–_d_*yz*_ orbitals, consistent with the behavior of Fe_3_GaTe_2_ (Figures S16 and S17).^19^ The enhanced magnitude of J_Fe1–Fe2_ in Fe_2_CoGaTe_2_ (56.8 meV) compared to Fe_3_GaTe_2_ (35.6 meV) is attributed to the increased magnetic moments of Fe_1_ and Fe_2_ atoms (2.59 μ_B_ and 2.54 μ_B_ in Fe_2_CoGaTe_2_ vs 2.35 μ_B_ and 2.31 μ_B_ in Fe_3_GaTe_2_). Additionally, the FM nature of J_Fe1–Co_ arises from the contribution of d_*x* ^2^–*y* ^2^–_d_*x* ^2^–*y* ^2^_, d_*x* ^2^–*y* ^2^_-d_*xy*_ and d_*xy*_-d_*xy*_ orbitals, with a more subtle effect of d_*z* ^2^–_d_*xz*_ and d_*z* ^2^_-d_*yz*_. In contrast, the AF behavior of J_Fe1–Fe1_ is mediated by the in-plane d_*x* ^2^–*y* ^2^_-d_*x* ^2^‑*y* ^2^_ and d_*xy*_-d_*xy*_ superexchange pathways, while antiferromagnetism in J_Co–Co_ mainly originates from a d_z2_–d_z2_ mechanism, mirroring the behavior of Fe_3_GeTe_2_.^19^ This contrasts with Fe_3_GaTe_2_, where the above-room-temperature ferromagnetism is stabilized by the combined FM character of interplane and in-plane exchange couplings (Figure S16). We attribute this to the different number of electrons in each material. Fe_3_GeTe_2_ contains one additional electron per Ge atom compared to Ga, and Fe_2_CoGaTe_2_ gains extra electrons from Co substitution relative to Fe_3_GaTe_2_. In both cases, the additional electrons stabilize AF in-plane interactions, leading to a reduction of the overall FM order and thus of the magnetic ordering temperature. Our computed T_N_ for twisted Fe_2_CoGaTe_2_ is 280 K (Figure S18). This value is expected to be overestimated, as our calculations yield T_N_ = 290 K for the untwisted bilayer Fe_2_CoGaTe_2_, higher than the experimental value of T_N_ ≈ 130 K.^25^ Such deviations are common in the determination of T_N_ and T_C_ in similar itinerant magnetic compounds.^57–59^ In particular, Fe_3_GaTe_2_ or Fe_3_GeTe_2_ have been reported to exhibit a mixed localized and itinerant magnetic character,^41,60^ which may require accounting for higher-order exchange terms, or explicitly itinerant effects for a fully quantitative description of the absolute ordering temperature.^57^ Nevertheless, our Heisenberg model correctly captures that the T_N_ in Fe_2_CoGaTe_2_ is reduced by a factor of ∼2 relative to the T_C_ of Fe_3_GaTe_2_, in good agreement with experimental findings (Figure S18).^24,25,39^

Given the interlayer antiferromagnetism and metallic character of Co-doped Fe_3_GaTe_2_, its potential for altermagnetism could be further explored through alternative strategies to realize this state in bulk (untwisted) Fe_3_GaTe_2_-based antiferromagnets. One possible approach is the realization of Janus configurations, where one Te layer is replaced by Se within each layer. Since the bulk unit cell consists of two layers, the resulting symmetry depends on whether the Se substitution occurs on the same or on opposite sides of the top and bottom layers. When the Janus asymmetry is applied on opposite sides of the two layers (i.e., the top side of one layer and the bottom side of the other layer), the system remains centrosymmetric (*P*3̅*m*1 (*D*_*3d*_)) and nonrelativistic spin splitting is forbidden. In contrast, if Se atoms are introduced on the same side of both layers, the inversion symmetry is broken, resulting in the noncentrosymmetric *P*6_3_mc (C_*6v*_) space group, which allows nonrelativistic spin splitting at zero net magnetization. Additionally, given that in bulk Fe_3‑x_GaTe_2_, Fe vacancies induce a symmetry lowering from *P*6_3_/*mmc* (*D*_*6h*_) to the polar *P*3*m*1 (*C*_*3v*_) space group due to displacement of central Fe atoms,^61–63^ a similar mechanism could be exploited via identical out-of-plane displacements of the central magnetic atoms to induce altermagnetism. Such displacements would break inversion symmetry, leading to the *P*6_3_mc (*C*_*6v*_) space group and enabling altermagnetism, whereas unequal displacements between central magnetic atoms of top and bottom layer would lower the symmetry to *P*3*m*1 (*C*_*3v*_), inducing a ferrimagnetic state. More broadly, the predicted altermagnetic state could be experimentally accessible through both spectroscopic and transport probes. In particular, ARPES and spin-resolved ARPES could directly resolve the calculated spin-split bands, following recent demonstrations in MnTe, CrSb, and KV_2_Se_2_O.^22,64–68^ Additionally, due to the breaking of P *T* symmetry in twisted Fe_2_CoGaTe_2_, nonzero anomalous magneto-optical and magneto-transport responses may be expected, including the magneto-optic Kerr effect (MOKE), giant magnetoresistance, and nonrelativistic spin-to-charge conversion.^7^

In conclusion, symmetry analysis reveals that twisted bilayer Fe_2_CoGaTe_2_ hosts an *i*-wave AM state in the nonrelativistic limit. This state arises from the interplay of AF interlayer coupling and the absence of inversion symmetry, thereby breaking the global P *T* symmetry. Our first first-principles calculations reveal a pronounced momentum-dependent spin splitting, reaching a maximum value of 138 meV, alongside with a spin-resolved Fermi surface exhibiting a set of star-like sheets arranged with 6-fold symmetry. Upon considering SOC effects, the spin degeneracy is preserved along the directions of the 2-fold rotation axes, but lifted along the perpendicular directions. Additionally, we provide a deep microscopic analysis of the magnetic exchange interactions governing the T_N_ in twisted bilayer Fe_2_CoGaTe_2_. We show that the additional electrons introduced by Co atoms drive a FM-to-AF transition of the in-plane exchange couplings relative to pristine Fe_3_GaTe_2_, and thus leading to a lower ordering temperature compared to the latter, mirroring the behavior of Fe_3_GeTe_2_. Overall, our results position twistronics as a versatile approach to induce altermagnetism in Fe_2_CoGaTe_2_, highlighting the potential of these techniques and related Fe-based metallic van der Waals antiferromagnets for their integration in high-efficiency spintronic devices.

## Methods

Calculations for bilayer Fe_3_GaTe_2_, Fe_2_CoGaTe_2_ and twisted Fe_2_CoGaTe_2_ were computed by employing the VASP package.^69^ We employ the generalized gradient approximation (GGA) to describe the exchange-correlation energy using a plane wave cutoff of 500 eV. Additionally, we employ the LDA functional to confirm the robustness of the interlayer AF coupling in twisted Fe_2_CoGaTe_2_. This choice is supported by previous studies showing that for Fe_3_GaTe_2_ and Fe_*n*_GeTe_2_ (*n* = 3, 4, 5), both GGA and LDA approaches accurately reproduce their electronic and magnetic properties.^19,70–73^ For the simulation of the bilayer structures, we included a vacuum spacing of 15 Å along the vertical direction to avoid spurious interactions with the periodic image. For treating the vdW interactions between layers, the DFT-D2 approximation was employed. Maximally localized Wannier functions were constructed using the Wannier90 package,^74^ for which the d orbitals of Fe and Co atoms as well as the p orbitals of Ga and Te were employed for the basis. The exchange couplings were obtained using of the TB2J code^75^ and the ordering temperature of the compounds through atomistic simulations as implemented in the VAMPIRE package.^76^

## Supplementary Material



## Notes

Bilayer Fe_3_GaTe_2_, bilayer Fe_2_CoGaTe_2_, twisted bilayer Fe_2_CoGaTe, twisted bilayer Fe_3_GaTe_2_, magnetic exchange couplings, and ordering temperature (PDF)
