# Supplementary material for: Twist-Induced Altermagnetism in a Metallic van der Waals Antiferromagnet
Source: Nano Lett. 2026 Jun 15;26(26):8513–21. doi: 10.1021/acs.nanolett.6c01391 (PMC13352942; doi:10.1021/acs.nanolett.6c01391)
Supplement: Supplementary file 1 [file nl6c01391_si_001.pdf]

Supporting Information

Twist-induced Altermagnetism in a Metallic van der Waals Antiferromagnet

Authors: Alberto M. Ruiz[1], Andrei Shumilin[1], Rafael González-Hernández[2], José J. Baldoví[1,*]

[1]Instituto de Ciencia Molecular, Universitat de València, Catedrático José Beltrán 2, 46980 Paterna, Spain.

[2]Departamento de Física y Geociencias, Universidad del Norte, Barranquilla, Colombia.

E-mail: j.jaime.baldovi@uv.es

Table of Contents

1. **Bilayer Fe_3GaTe_2** **2**

2. **Bilayer $Fe_2CoGaTe_2$** **3**

3. **Twisted bilayer $Fe_2CoGaTe_2$** **5**

   3.1 **Atomic coordinates of 21.79º twisted bilayer $Fe_2CoGaTe_2$** 5

   3.2 **Symmetry operations leading to *i*-wave altermagnetic state in 21.79º twisted bilayer $Fe_2CoGaTe_2$** 7

   3.3 **Additional twist angles leading to *i*-wave altermagnetism** 8

   3.4 **Interplay between SOC and altermagnetism** 11

   3.5 **Impact of chemical Fe/Co disorder on altermagnetism** 13

4. **Twisted bilayer Fe_3GaTe_2** **16**

5. **Magnetic exchange couplings and ordering temperature** **17**

1. Bilayer Fe_3GaTe_2

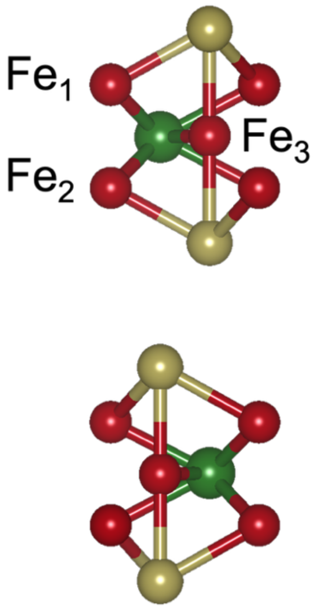

Figure S1. Lateral view of bilayer Fe_3GaTe_2. Color code: Fe (dark red), Ga (green), and Te (yellow).

Table S1. Magnetic moments for Fe_1, Fe_2 and Fe_3 atoms for bilayer Fe_3GaTe_2.

Atom	Magnetic moment (μ_B)
Fe_1	2.347
Fe_2	2.310
Fe_3	1.423
Average	2.026

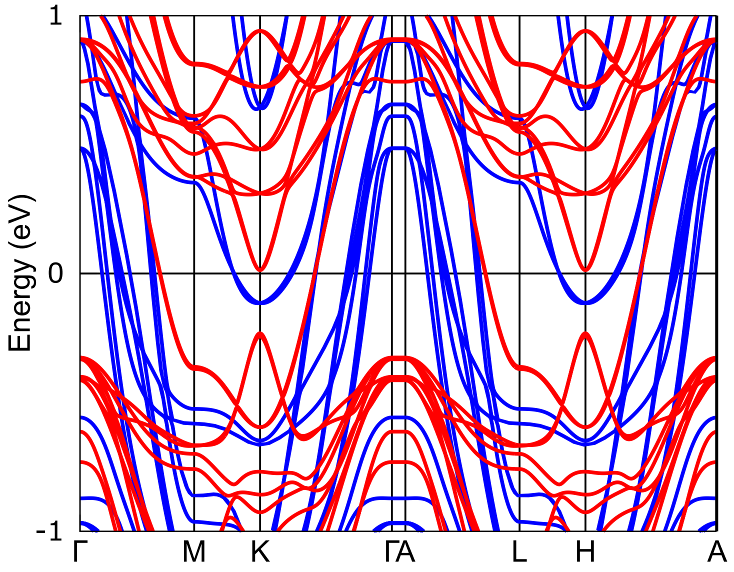

Figure S2. Band structure of bilayer Fe_3GaTe_2. Blue (red) color in the band structure indicates spin up (down) states.

2. Bilayer $Fe_2CoGaTe_2$

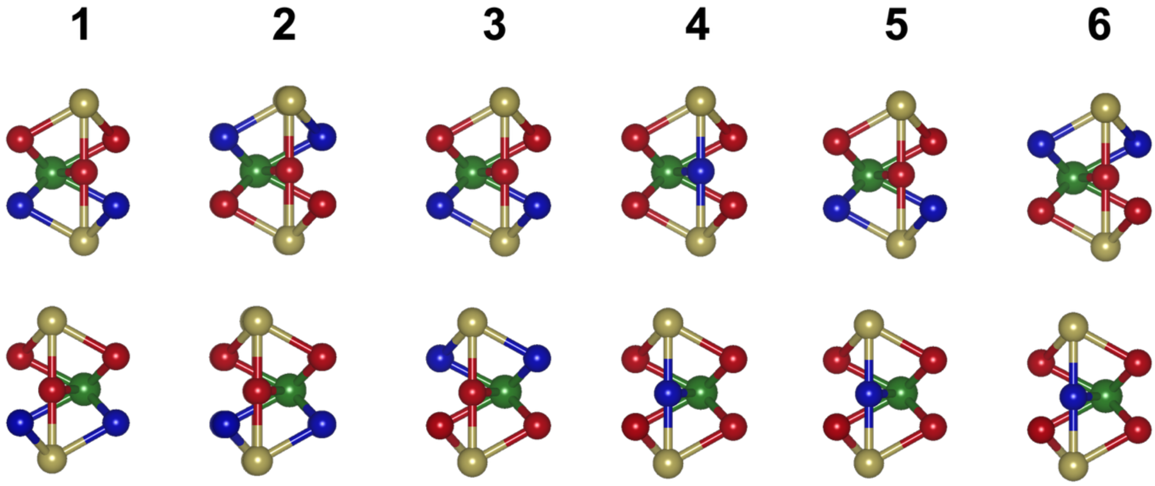

Figure S3. Lateral views of bilayer $Fe_2CoGaTe_2$, showing the six symmetry-inequivalent configurations corresponding to different Co atomic positions.

Table S2. Total energies of the six possible Co atom arrangements in bilayer $Fe_2CoGaTe_2$, expressed relative to the ground state configuration.

Configuration	Total energy (meV)
1	362.97
2	395.67
3	329.82
4	0.00
5	164.14
6	197.25

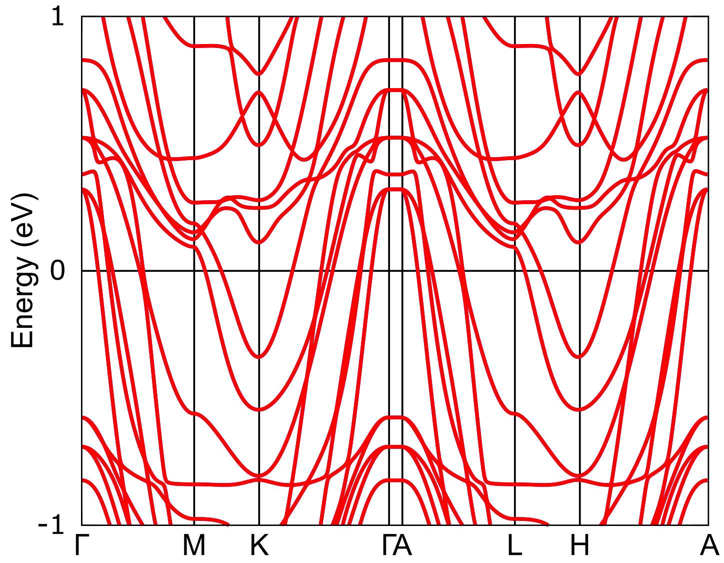

Figure S4. Band structure of bilayer $Fe_2CoGaTe_2$ showing AF character given that spin up (blue) and down (red) states are overlapped.

3. Twisted bilayer $Fe_2CoGaTe_2$

3.1 Atomic coordinates of 21.79° twisted bilayer $Fe_2CoGaTe_2$

Table S3. Atomic coordinates of the 21.79° twisted $Fe_2CoGaTe_2$ bilayer structure.

```
Fe Co Ga Te
   1.0000000000000000
  -10.1223755121422734    3.5064937360460924    0.0000000000000000
    2.0244751024284549  -10.5194812081382771    0.0000000000000000
    0.0000000000000000    0.0000000000000000   29.4385391698342502
  Fe   Co   Ga   Te
   28    14    14    28
Direct
  0.0000000000000000  0.0000000000000000  0.0889819581463678
  0.8571428571428541  0.2857142857142847  0.0886366504891050
  0.7142857142857153  0.5714285714285694  0.0886366504891050
  0.5714285714285694  0.8571428571428541  0.0888499206784472
  0.4285714285714306  0.1428571428571459  0.0886366504891050
  0.2857142857142847  0.4285714285714306  0.0888499206784472
  0.1428571428571459  0.7142857142857153  0.0888499206784472
  0.0000000000000000  0.0000000000000000  0.1717100839798023
  0.8571428571428541  0.2857142857142847  0.1713663094372500
  0.7142857142857153  0.5714285714285694  0.1713663094372500
  0.5714285714285694  0.8571428571428541  0.1715019677942475
  0.4285714285714306  0.1428571428571459  0.1713663094372500
  0.2857142857142847  0.4285714285714306  0.1715019677942475
  0.1428571428571459  0.7142857142857153  0.1715019677942475
  0.0000000000000000  0.0000000000000000  0.3698861050201940
  0.8571428571428541  0.5714285714285694  0.3702298795627461
  0.5714285714285694  0.7142857142857153  0.3700942212057489
  0.2857142857142847  0.8571428571428541  0.3700942212057489
  0.7142857142857153  0.1428571428571459  0.3702298795627461
  0.4285714285714306  0.2857142857142847  0.3702298795627461
  0.1428571428571459  0.4285714285714306  0.3700942212057489
  0.0000000000000000  0.0000000000000000  0.4526142298536319
  0.8571428571428541  0.5714285714285694  0.4529595375108946
  0.5714285714285694  0.7142857142857153  0.4527462673215527
  0.2857142857142847  0.8571428571428541  0.4527462673215527
  0.7142857142857153  0.1428571428571459  0.4529595375108946
  0.4285714285714306  0.2857142857142847  0.4529595375108946
  0.1428571428571459  0.4285714285714306  0.4527462673215527
  0.9523809523809490  0.7619047619047592  0.1298125448416617
  0.8095238095238102  0.0476190476190510  0.1298125448416617
  0.6666666666666643  0.3333333333333357  0.1284732934287333
  0.5238095238095255  0.6190476190476204  0.1297589707625481
  0.3809523809523796  0.9047619047619051  0.1297589707625481
  0.2380952380952408  0.1904761904761898  0.1298125448416617
  0.0952380952380949  0.4761904761904745  0.1297589707625481
  0.8095238095238102  0.7619047619047592  0.4117836431583379
  0.5238095238095255  0.9047619047619051  0.4118372172374516
  0.9523809523809490  0.1904761904761898  0.4117836431583379
  0.6666666666666643  0.3333333333333357  0.4131228945712663
  0.3809523809523796  0.4761904761904745  0.4118372172374516
  0.0952380952380949  0.6190476190476204  0.4118372172374516
  0.2380952380952408  0.0476190476190510  0.4117836431583379
  0.9047619047619051  0.5238095238095255  0.1305481467728052
  0.7619047619047592  0.8095238095238102  0.1307029423885822
  0.6190476190476204  0.0952380952380949  0.1305481467728052
  0.4761904761904745  0.3809523809523796  0.1305481467728052
```

0.3333333333333357	0.6666666666666643	0.1306430818159592
0.1904761904761898	0.9523809523809490	0.1307029423885822
0.0476190476190510	0.2380952380952408	0.1307029423885822
0.7619047619047592	0.9523809523809490	0.4108932456114175
0.9047619047619051	0.3809523809523796	0.4110480412271945
0.6190476190476204	0.5238095238095255	0.4110480412271945
0.3333333333333357	0.6666666666666643	0.4109531061840405
0.0476190476190510	0.8095238095238102	0.4108932456114175
0.4761904761904745	0.0952380952380949	0.4110480412271945
0.1904761904761898	0.2380952380952408	0.4108932456114175
0.9523809523809490	0.7619047619047592	0.0443073841659125
0.8095238095238102	0.0476190476190510	0.0443073841659125
0.6666666666666643	0.3333333333333357	0.0434217526129136
0.5238095238095255	0.6190476190476204	0.0442676298820156
0.3809523809523796	0.9047619047619051	0.0442676298820156
0.2380952380952408	0.1904761904761898	0.0443073841659125
0.0952380952380949	0.4761904761904745	0.0442676298820156
0.9523809523809490	0.7619047619047592	0.2156404961107135
0.8095238095238102	0.0476190476190510	0.2156404961107135
0.6666666666666643	0.3333333333333357	0.2141762677004304
0.5238095238095255	0.6190476190476204	0.2155579177279404
0.3809523809523796	0.9047619047619051	0.2155579177279404
0.2380952380952408	0.1904761904761898	0.2156404961107135
0.0952380952380949	0.4761904761904745	0.2155579177279404
0.8095238095238102	0.7619047619047592	0.3259556928892829
0.5238095238095255	0.9047619047619051	0.3260382712720560
0.9523809523809490	0.1904761904761898	0.3259556928892829
0.6666666666666643	0.3333333333333357	0.3274199212995658
0.3809523809523796	0.4761904761904745	0.3260382712720560
0.0952380952380949	0.6190476190476204	0.3260382712720560
0.2380952380952408	0.0476190476190510	0.3259556928892829
0.8095239047619032	0.7619047142857127	0.4972888038340871
0.5238096190476185	0.9047618571428586	0.4973285581179842
0.9523810476190491	0.1904761428571433	0.4972888038340871
0.6666667619047644	0.3333332857142892	0.4981744353870862
0.3809524761904726	0.4761904285714280	0.4973285581179842
0.0952381904761879	0.6190475714285739	0.4973285581179842
0.2380953333333338	0.0476189999999974	0.4972888038340871

3.2 Symmetry operations leading to *i*-wave altermagnetic state in 21.79° twisted bilayer $Fe_2CoGaTe_2$

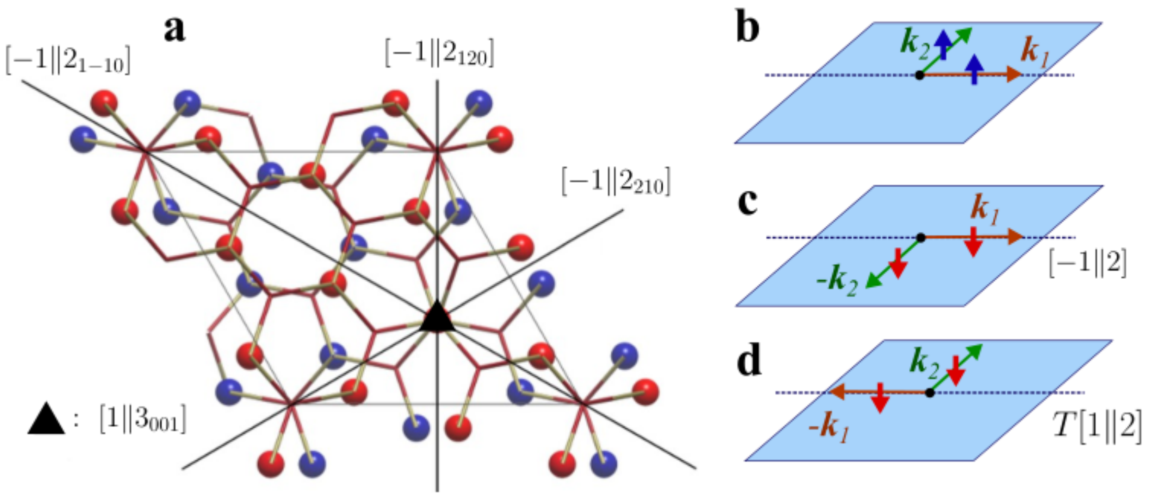

Figure S5 a) Illustration of the symmetries in the 21.79° twisted $Fe_2CoGaTe_2$ bilayer. b-d) Illustration of the action of a $[-1 \parallel 2]$ and $T \cdot [1 \parallel 2]$ symmetries on an electron propagating parallel and perpendicular to an in-plane twofold rotation axis, with wavevectors $k_1$and k_2, respectively. Spin-up and spin-down states are indicated by blue and red arrows. b) Initial electron states, c) the electron states after application of $[-1 \parallel 2]$ operation, d) the states after application of $T \cdot [1 \parallel 2]$ operation.

The crystal structure of the 21.79° twisted bilayer $Fe_2CoGaTe_2$ belongs to the P312 (No. 149) space group. It contains a single threefold rotation axis perpendicular to the bilayer plane and three in-plane twofold rotation axes, as shown in Figure S5(a). Below the Néel temperature, time-reversal symmetry is spontaneously broken. Consequently, the twofold rotations—when acting solely on the crystal structure—must be combined with a spin-flip operation, giving rise to the non-relativistic symmetries $[-1 \parallel 2_{1-10}]$, $[-1 \parallel 2_{120}]$, and $[-1 \parallel 2_{210}]$. In the non-relativistic limit, any collinear magnetic system also possesses the symmetry $T \cdot [-1 \parallel 1]$, which combines a spin-flip with time-reversal symmetry T.[1] This operation acts on both spin and electron momentum yielding three additional symmetries $T \cdot [1 \parallel 2_{1-10}]$, $T \cdot [1 \parallel 2_{120}]$, and $T \cdot [1 \parallel 2_{210}]$. Together, these six symmetries give rise to i-wave altermagnetism and enforce electron-state degeneracies along six high-symmetry directions. As illustrated in Figure S5(b–d), each $[-1 \parallel 2]$ symmetry guarantees spin degeneracy along the corresponding twofold rotation axis, while the associated $T \cdot [1 \parallel 2]$ symmetry protects degeneracy along the direction perpendicular to that axis.

3.3 Additional twist angles leading to *i*-wave altermagnetism

Table S4 summarizes other representative commensurate twist angles of twisted $Fe_2CoGaTe_2$ bilayers and the number of atoms within the unit cell. In all cases, the structures preserve in-plane C_2 rotations connecting opposite-spin sublattices and C_{3z} symmetry within each sublattice, leading to a *i*-wave altermagnetic state (Figure S6). The corresponding band structure for each case confirms the presence of nonrelativistic spin splitting (Figure S7).

Table S4. Different twist angles for bilayer $Fe_2CoGaTe_2$ along with the number of atoms considered within the unit cell and their corresponding lattice parameters and angles.

Rotation (°)	Number of atoms	$a = b$ (Å)	α, β, γ (°)
13.17	228	17.65	90, 90, 120
21.79	84	10.71	90, 90, 120
27.79	156	14.60	90, 90, 120
32.21	156	14.60	90, 90, 120
38.21	84	10.71	90, 90, 120
46.83	228	17.65	90, 90, 120

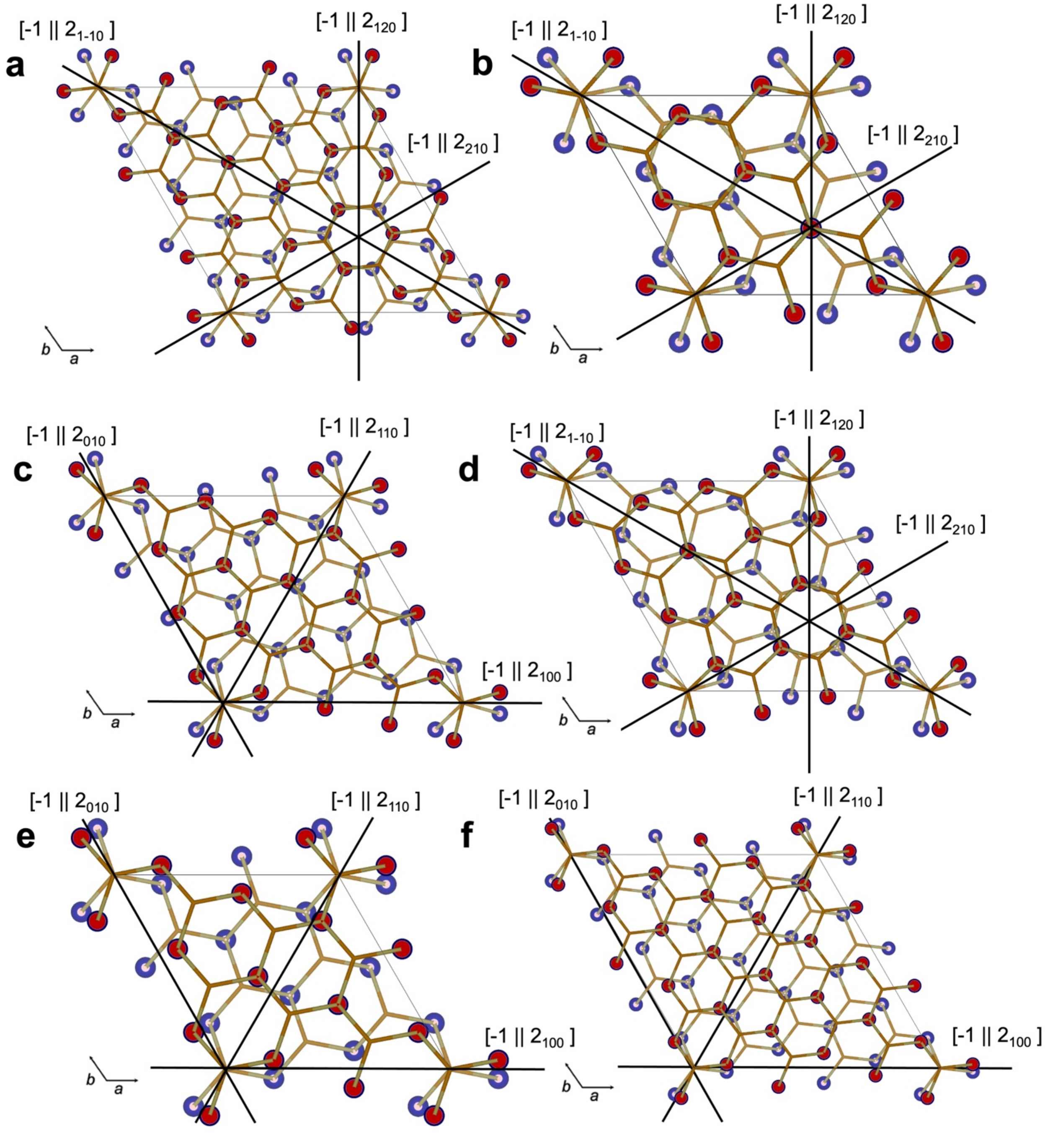

Figure S6. Top views of twisted bilayer $Fe_2CoGaTe_2$ for twist angles a) 13.17º, b) 21.79º, c) 27.79º, d) 32.21º, e) 38.21º and f) 46.83º. Here, blue and red balls represent Co atoms from the bottom and top layer, respectively. For clarity, we only represent Co atoms of the material. Additionally, we show the symmetry operations relating opposite Co spin sublattices.

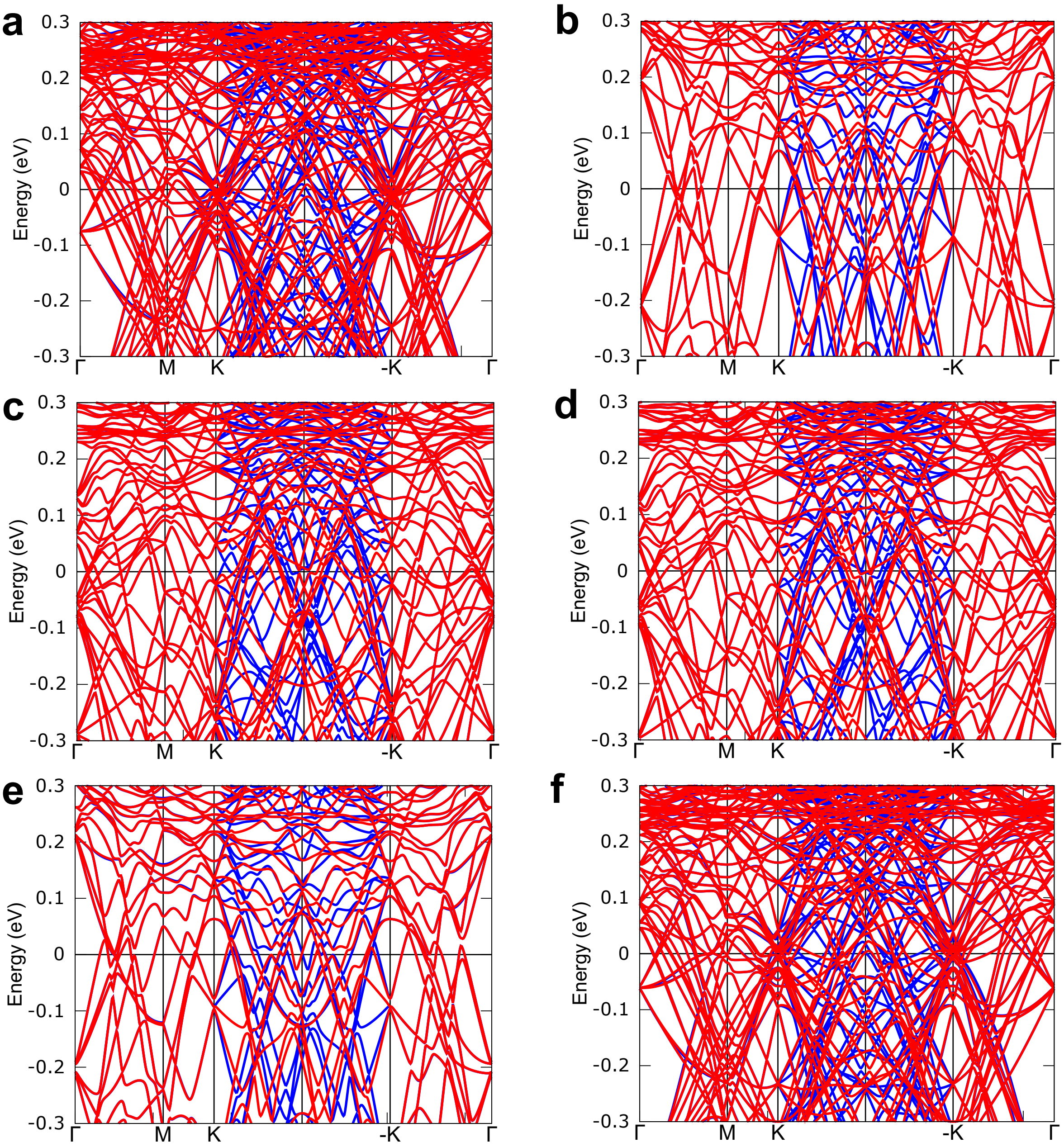

Figure S7. Nonrelativistic electronic band structure of twisted bilayer $Fe_2CoGaTe_2$ along the high-symmetry path Γ−M−K−(−)K− Γ for twist angles a) 13.17°, b) 21.79°, c) 27.79°, d) 32.21°, e) 38.21° and f) 46.83°. Blue (red) color in the band structure indicates spin up (down) states.

3.4 Interplay between SOC and altermagnetism

When SOC is included, the symmetries of the antiferromagnetic bilayer are described by the magnetic space group #149.21. In this case, the twofold rotations act simultaneously on spatial coordinates and spin directions, without the need for an additional time-reversal operation. This symmetry enforces spin degeneracy along the rotation axis and imposes constraints on the allowed spin-polarization directions. Specifically, when a twofold rotation C_2 is applied to an electron state with wavevector $\mathbf{k}$ and spin polarization $\mathbf{s}$, symmetry requires the existence of another electron state at $\mathbf{k}' = C_2\mathbf{k}$ with the same energy and spin polarization $C_2\mathbf{s}$. In the special case of non-degenerate states with wavevectors $\mathbf{k}_1$ lying along the rotation axis, one has $\mathbf{k}_1' = \mathbf{k}_1$, and the C_2 operation must therefore conserve the spin-polarization direction (see Figure S8(a)). This symmetry forbids spin polarization perpendicular to the rotation axis, and in particular along the out-of-plane direction. As a result, the electronic band structure along the directions of the twofold rotation axes remains nearly degenerate, even when SOC is included.

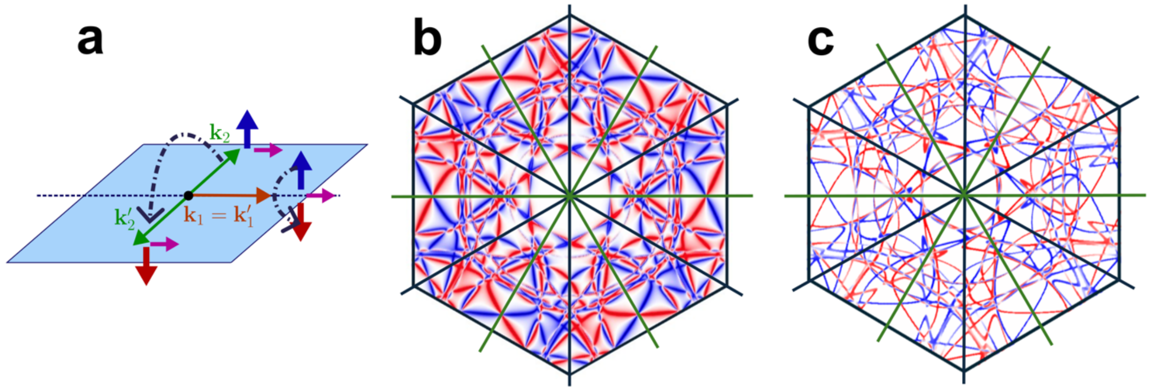

Figure S8. a) Schematic illustration of the magnetic two-fold rotation acting on the wavevectors $\boldsymbol{k}_1$and $\boldsymbol{k}_2$, following the notation of Figure S5. The operation reverses the out-of-plane (z) spin component (blue-to-red arrow) while preserving the spin component along the rotation axis (magenta arrow). When the electron wavevector is conserved during the rotation ($\boldsymbol{k}_1 = \boldsymbol{k}_1'$), only the spin component parallel to the rotation axis is allowed for non-degenerate states. b) Fermi surface of the twisted $Fe_2CoGaTe_2$ bilayer calculated without spin–orbit coupling (SOC). c) Fermi surface of the twisted $Fe_2CoGaTe_2$ bilayer calculated with SOC (polarization in z-direction is shown with red and blue colours). In panels b) and c), the green lines indicate the directions of the twofold symmetry axes, while the black lines denote the perpendicular directions.

In Figure S8(b,c) we show that the states at the Fermi level calculated for the twisted $Fe_2CoGaTe_2$ bilayer without and with SOC, respectively. Consistent with our symmetry analysis, the states are spin-degenerate along all six directions in the absence of SOC. When SOC is included, this degeneracy is preserved along the directions of the twofold rotation axes (indicated by green lines), but is lifted along the perpendicular directions (black lines), where no symmetry protection is provided by the magnetic space group #149.21.

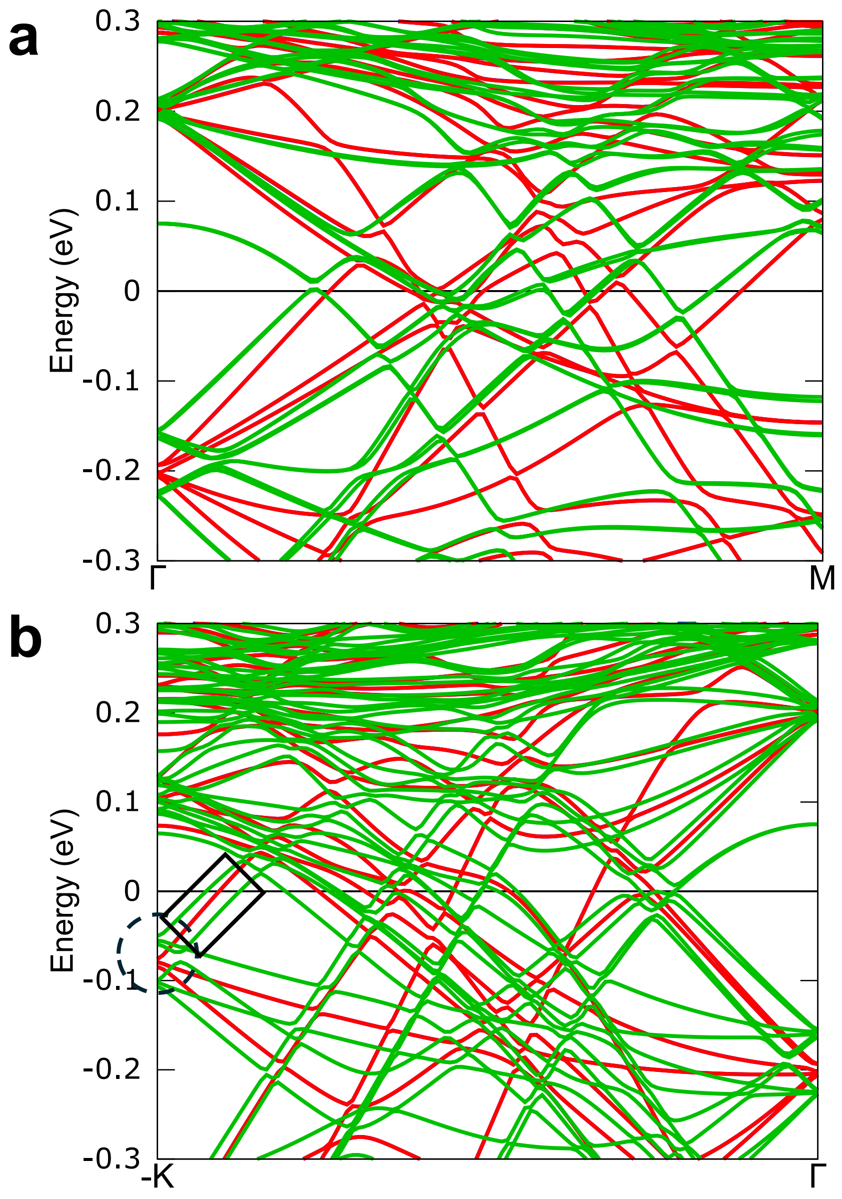

Figure S9. Electronic band structure of 21.79º twisted $Fe_2CoGaTe_2$ without (red) and with (green) SOC along a) Γ–M and b) (−)K–Γ directions. The highlighted regions show the spin splitting due to the effect of SOC.

Note that the effect of SOC is strongly anisotropic. In particular, along Γ–M the spin degeneracy is preserved due to magnetic symmetry protection, while along (−)K–Γ SOC lifts the degeneracy of several bands (highlighted), leading to splitting of up to ~45 meV near the Fermi level. In both directions, SOC also induces an energy shift due to the strong spin-orbit character of Te atoms.

3.5 Impact of chemical Fe/Co disorder on altermagnetism

Chemical disorder was simulated by randomly interchanging Fe and Co atoms at different concentrations (9.52%, 19.1%, and 47.6%). For each disorder level, several configurations were considered to account for statistical variability.

We find that at low disorder (~10%), the system preserves some altermagnetic features, with momentum-dependent nonrelativistic spin splitting still visible along the K−(−)K direction (Figure S10). At intermediate disorder (~20%), the global magnetic order remains compensated, but the altermagnetism is significantly reduced, and only partial signatures persist (Figure S11). At high disorder (~50%), the altermagnetic character is completely suppressed (Figure S12), and the system evolves toward a compensated ferrimagnetic state (Table S5).

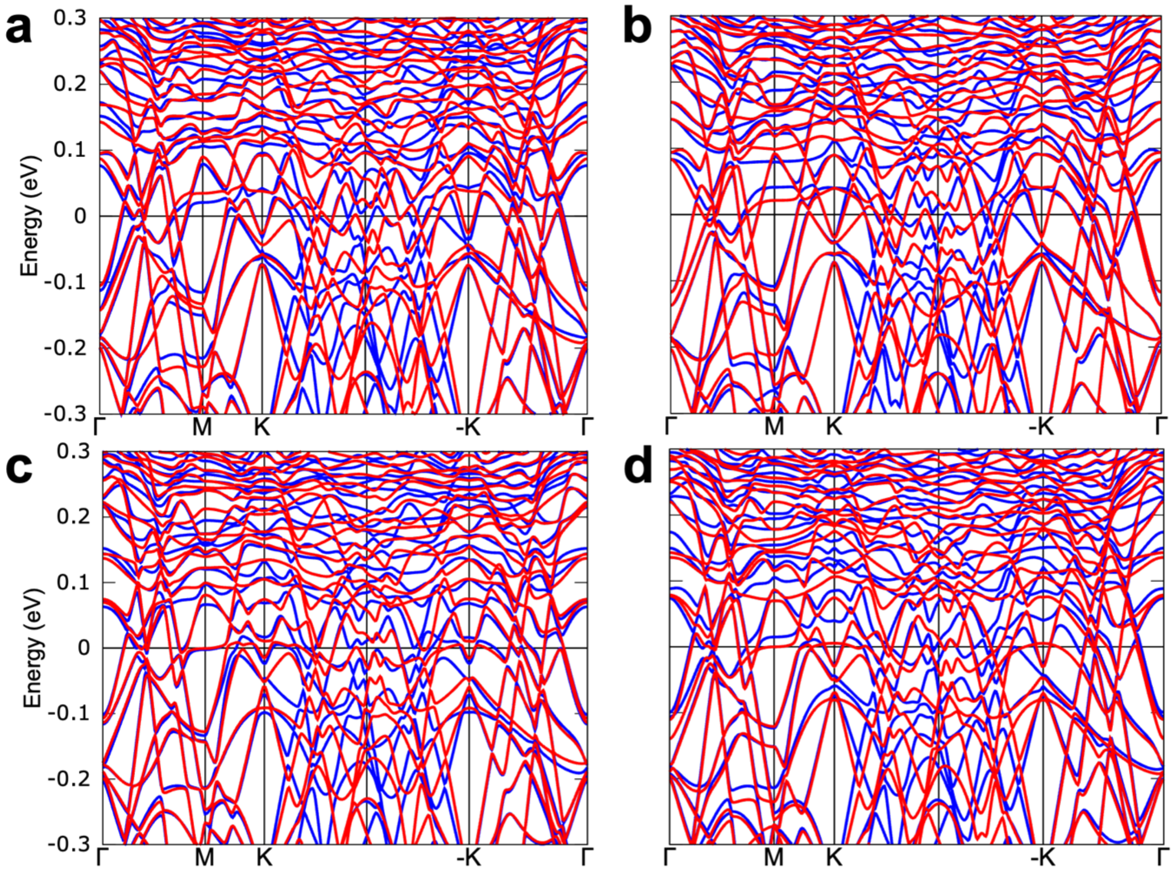

Figure S10. Electronic band structures of twisted $Fe_2CoGaTe_2$ with 9.52% Fe/Co disorder for four representative configurations, namely a) configuration 1, b) configuration 2, c) configuration 3, and d) configuration 4. Blue (red) color in the band structure indicates spin up (down) states.

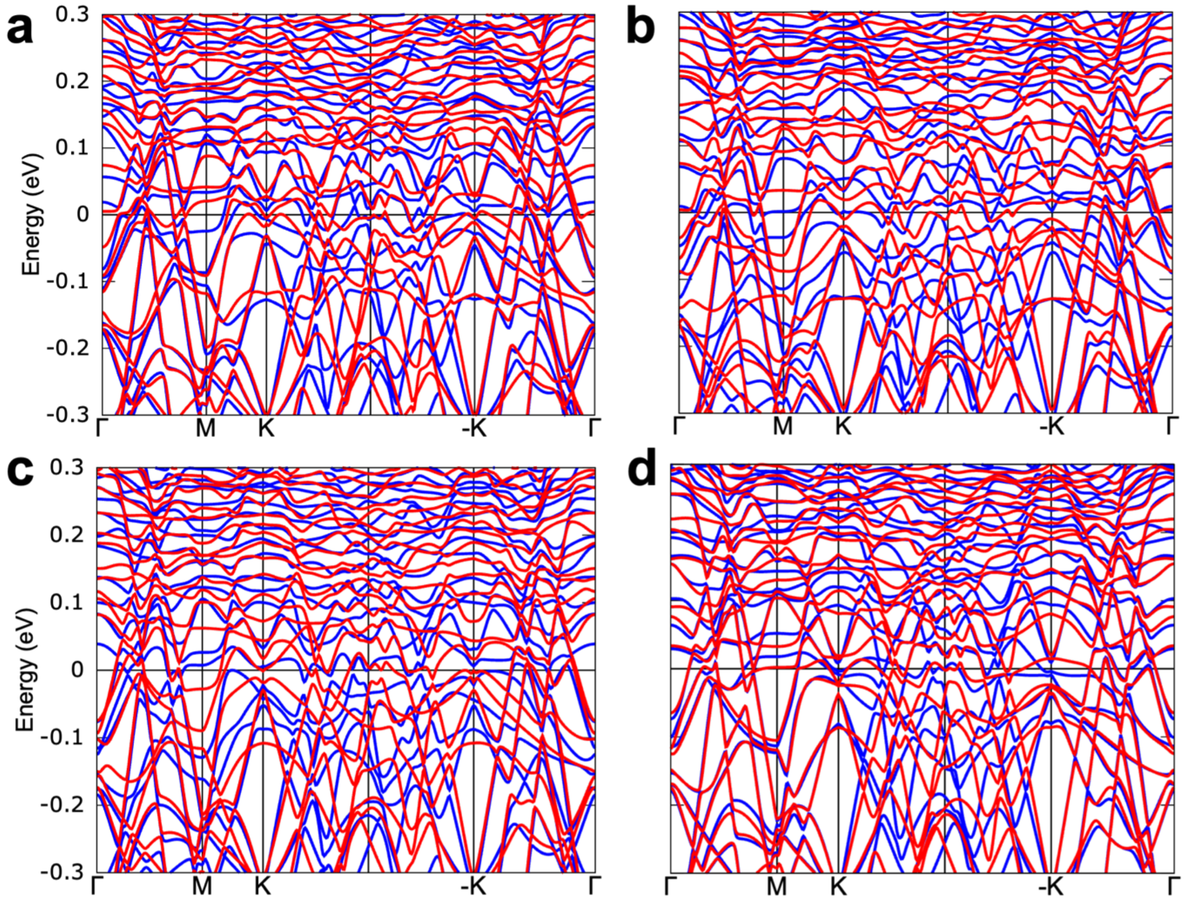

Figure S11. Electronic band structures of twisted $Fe_2CoGaTe_2$ with 19.1% Fe/Co disorder for four representative configurations, namely a) configuration 1, b) configuration 2, c) configuration 3, and d) configuration 4. Blue (red) color in the band structure indicates spin up (down) states.

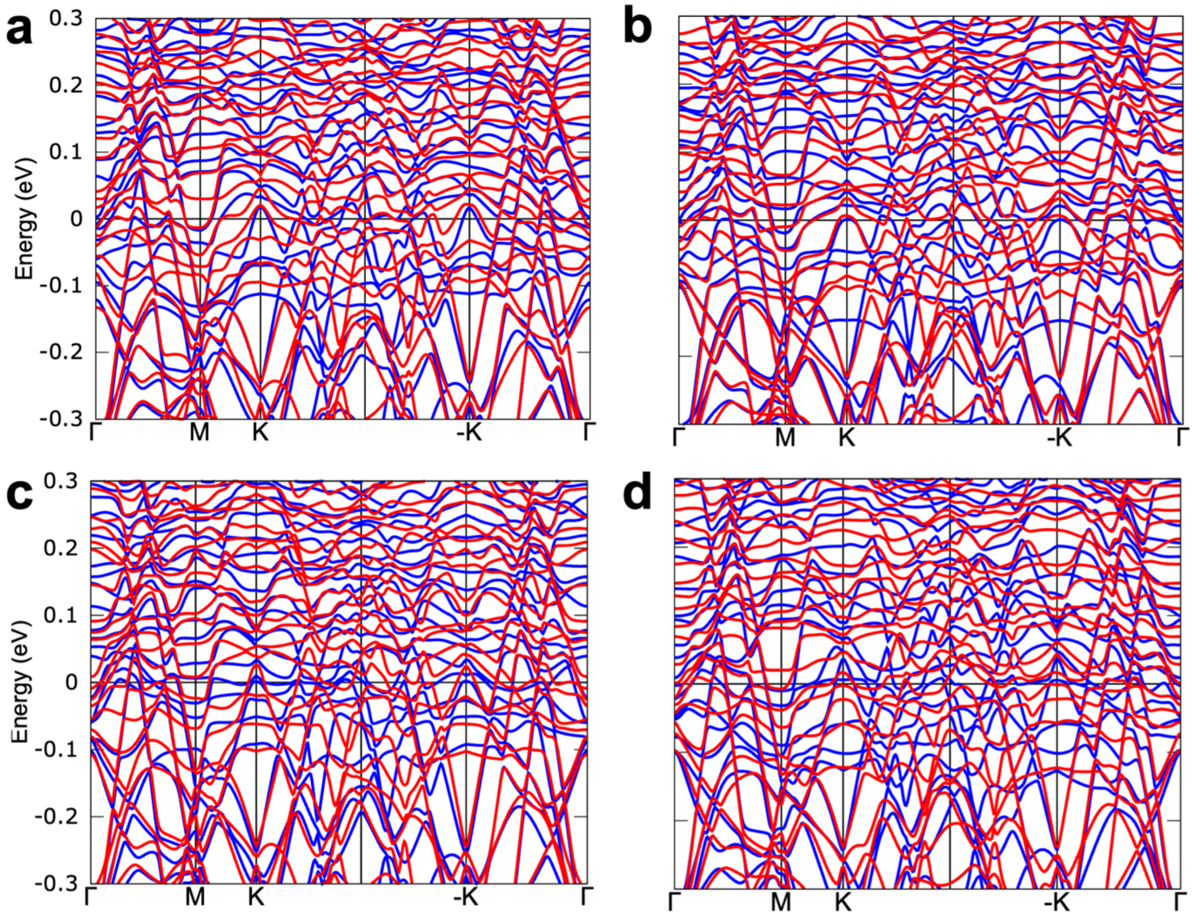

Figure S12. Electronic band structures of twisted $Fe_2CoGaTe_2$ with 47.6% Fe/Co disorder for four representative configurations, namely a) configuration 1, b) configuration 2, c) configuration 3, and d) configuration 4. Blue (red) color in the band structure indicates spin up (down) states.

Table S5. Total energy and global magnetic moment as a function of disorder for 21.79º twisted $Fe_2CoGaTe_2$.

Disorder (%)	Configuration	Total energy (meV)	Total Magnetic moment (μ_B/unit cell)
0	Pristine	0.00	0.00
9.52	1	460.94	0.29
	2	506.85	0.00
	3	484.37	0.04
	4	469.00	-0.20
19.1	1	918.83	0.20
	2	876.18	0.30
	3	875.84	0.20
	4	966.58	0.00
47.6	1	900.98	0.15
	2	2212.07	0.04
	3	2197.03	0.21
	4	2221.90	0.33

4. Twisted bilayer Fe_3GaTe_2

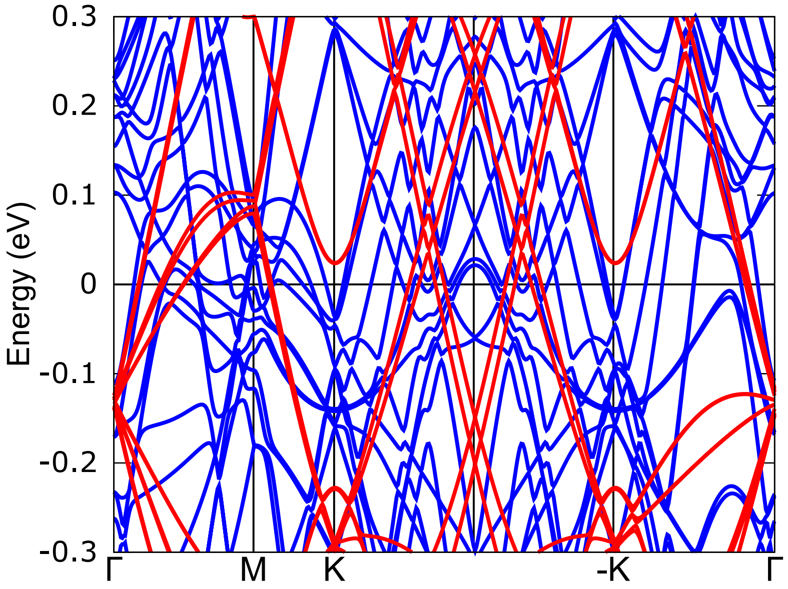

Figure S13. Band structure of twisted bilayer Fe_3GaTe_2 along the high-symmetry path Γ−M−K−(−)K−Γ. Blue (red) color in the band structure indicates spin up (down) states.

5. Magnetic exchange couplings and ordering temperature

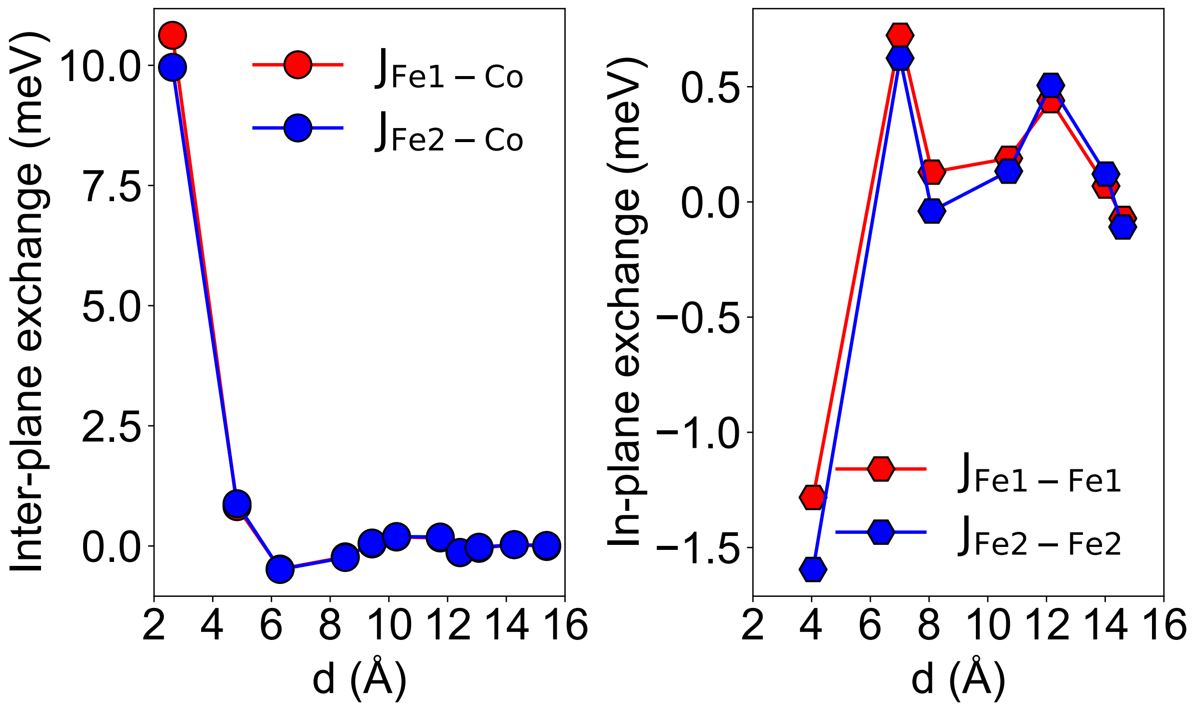

Figure S14. Comparison between J_{Fe1-Co} and J_{Fe2-Co} (left) as well as between $J_{Fe1-Fe1}$ and $J_{Fe2-Fe2}$ (right) for twisted bilayer $Fe_2CoGaTe_2$.

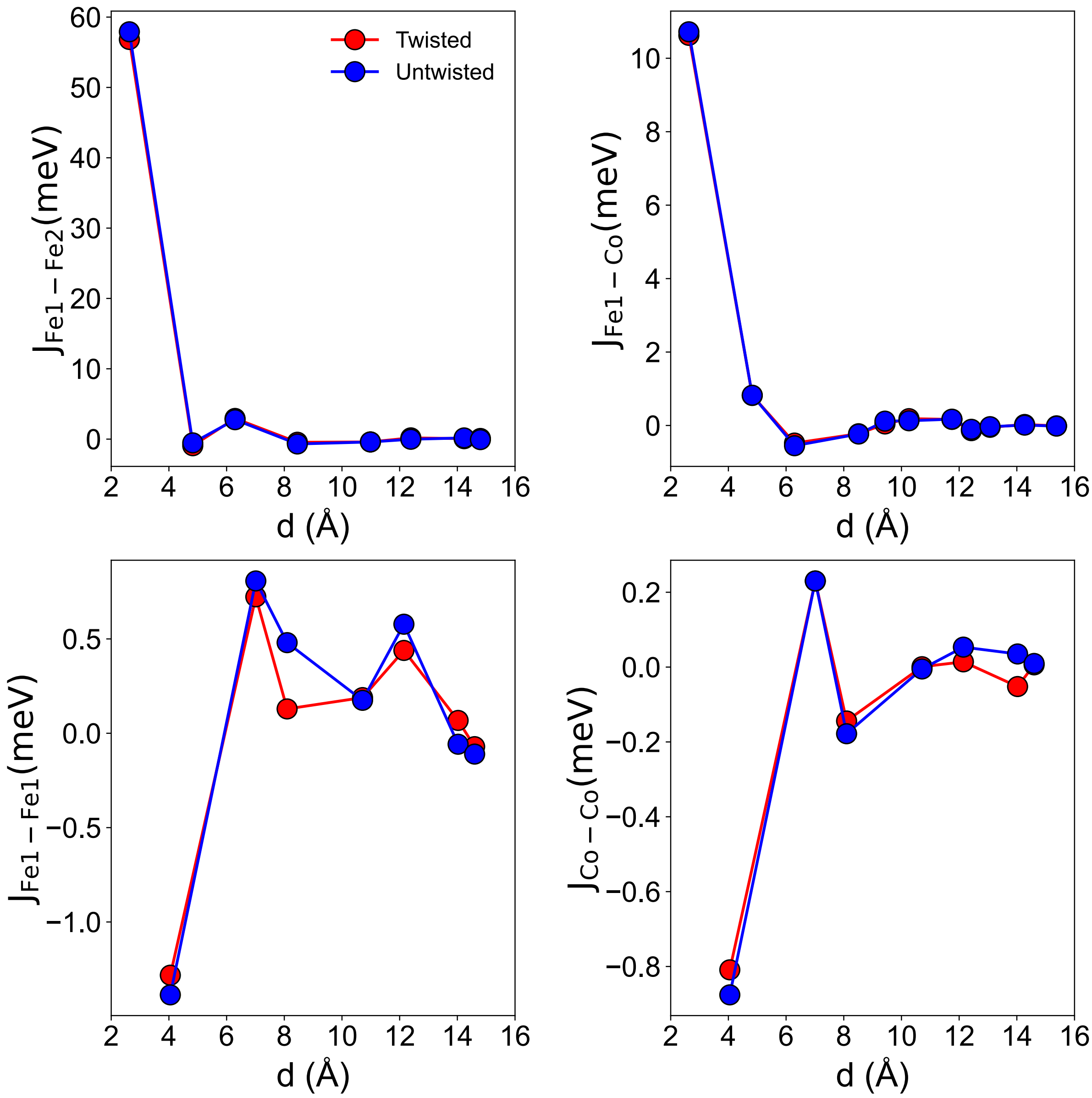

Figure S15. Comparison of the exchange couplings $J_{Fe1\text{-}Fe2}$, $J_{Fe1\text{-}Co}$, $J_{Fe1\text{-}Fe1}$ and $J_{Co\text{-}Co}$ between twisted and untwisted $Fe_2CoGaTe_2$.

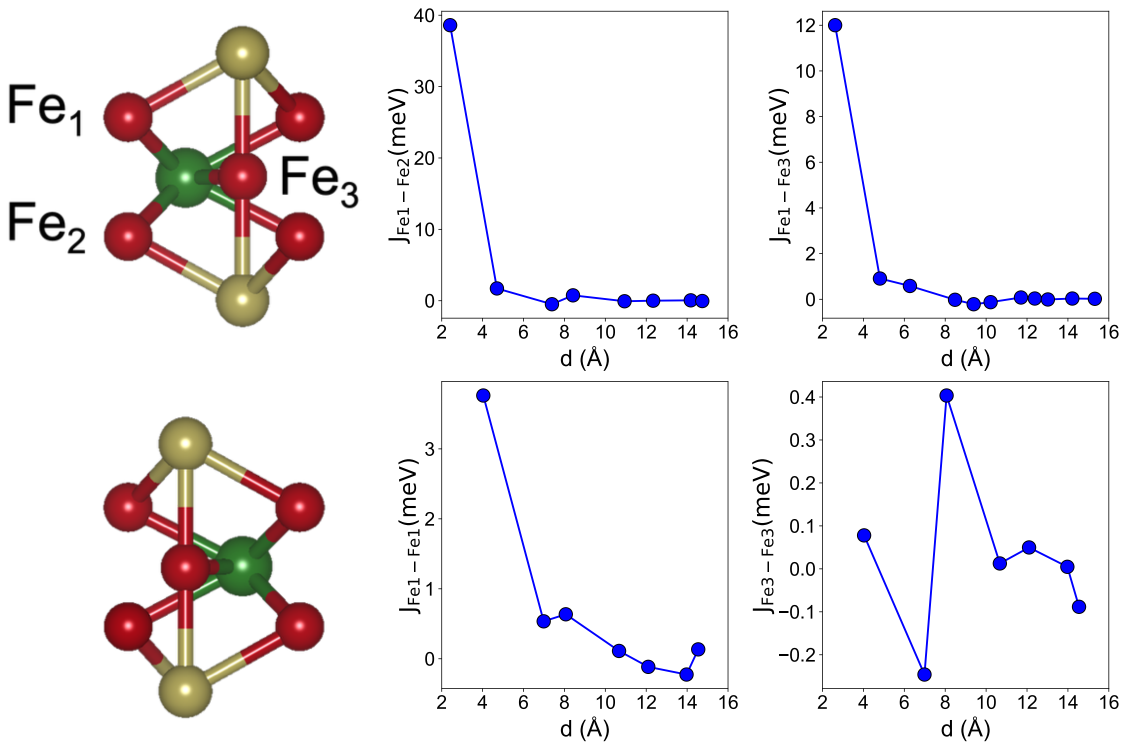

Figure S16. Exchange couplings $J_{Fe1\text{-}Fe2}$, $J_{Fe1\text{-}Fe3}$, $J_{Fe1\text{-}Fe1}$ and $J_{Fe3\text{-}Fe3}$ and its evolution with distance for bilayer Fe_3GaTe_2.

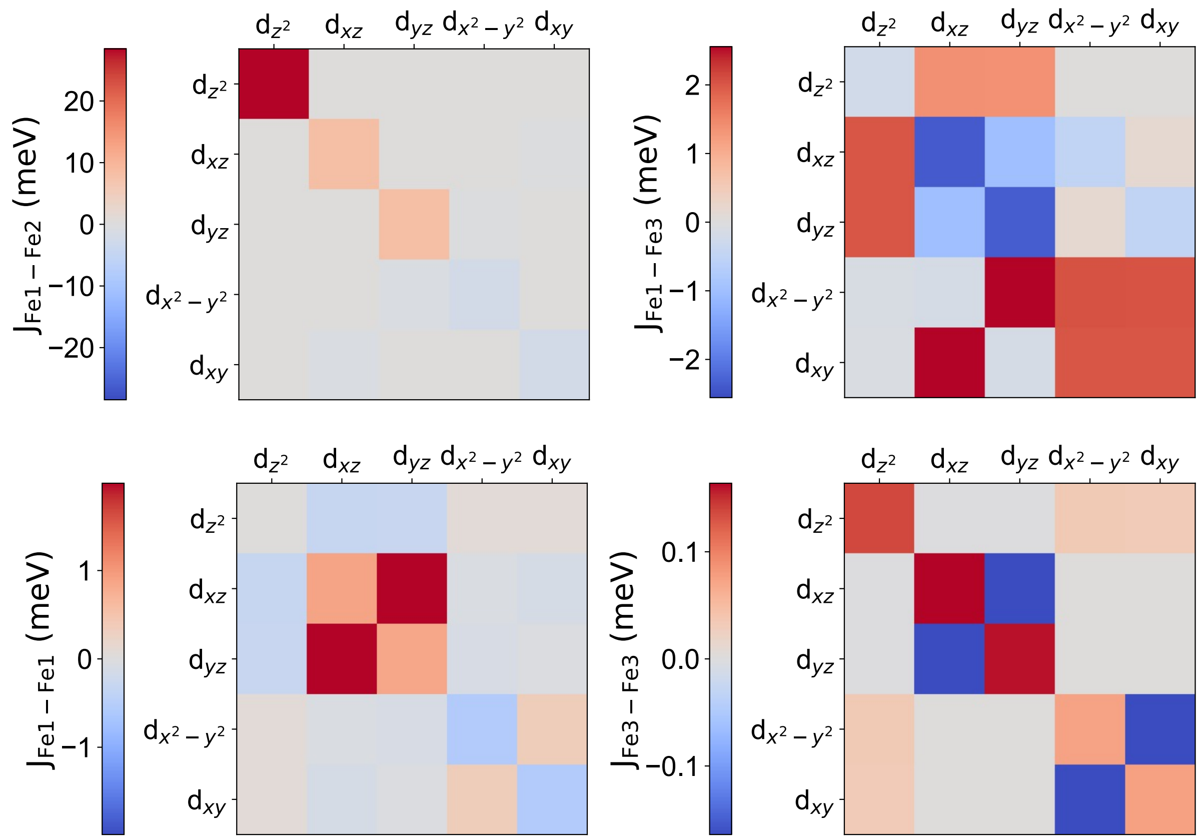

Figure S17. Orbital-resolved contribution to $J_{Fe1\text{-}Fe2}$, $J_{Fe1\text{-}Fe3}$, $J_{Fe1\text{-}Fe1}$ and $J_{Fe3\text{-}Fe3}$ for bilayer Fe_3GaTe_2.

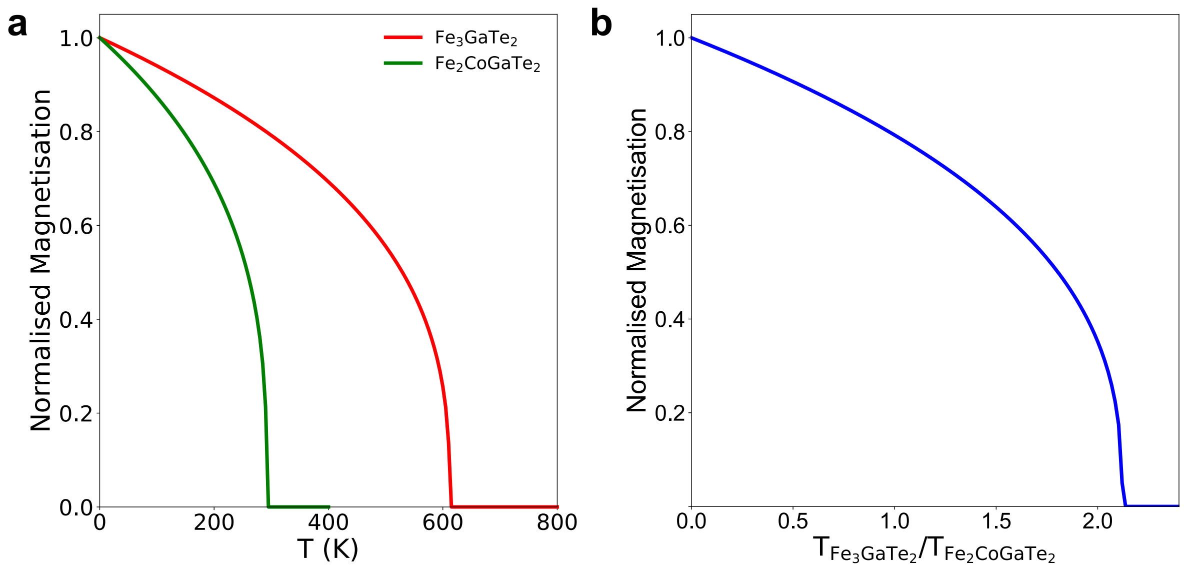

Figure S18. a) Evolution of normalised magnetization as a function of temperature for bilayer Fe_3GaTe_2 (T_C = 620 K) and $Fe_2CoGaTe_2$ (T_N = 292 K). b) Ratio of the magnetic ordering temperatures of bilayer Fe_3GaTe_2 and $Fe_2CoGaTe_2$.

Figure S18 shows that the calculated T_N of bilayer $Fe_2CoGaTe_2$ is 292 K, which overestimates the experimental value of T_N = 130 K. For bilayer Fe_3GaTe_2, we find that T_C = 620 K, higher than the experimental value of 290 K. Despite the overestimation of the absolute value of magnetic ordering temperatures, the calculated ratio of $T_{Fe3GaTe2}/T_{Fe2CoGaTe2}$ = 2.12 is in good agreement with experimental observations.[2,3]

REFERENCES

(1) Liu, Y.; Yu, J.; Liu, C.-C. Twisted Magnetic Van Der Waals Bilayers: An Ideal Platform for Altermagnetism. *Phys. Rev. Lett.* **2024**, *133* (20), 206702.

(2) Wang, M.; Lei, B.; Zhu, K.; Deng, Y.; Tian, M.; Xiang, Z.; Wu, T.; Chen, X. Hard Ferromagnetism in van Der Waals Fe_3GaTe_2 Nanoflake down to Monolayer. *NPJ 2D Mater. Appl.* **2024**, *8* (1), 22.

(3) Wang, M.; Zhu, K.; Lei, B.; Deng, Y.; Hu, T.; Song, D.; Du, H.; Tian, M.; Xiang, Z.; Wu, T.; Chen, X. Layer-Number-Dependent Magnetism in the Co-Doped van Der Waals Ferromagnet Fe_3GaTe_2. *Nano Lett.* **2024**, *24* (14), 4141–4149.
